# GICEDCam: A Geospatial Internet of Things Framework for Complex Event Detection in Camera Streams

**DOI:** 10.3390/s25175331

**Published:** 2025-08-27

**Authors:** Sepehr Honarparvar, Yasaman Honarparvar, Zahra Ashena, Steve Liang, Sara Saeedi

**Affiliations:** 1 Department of Geomatics Engineering, University of Calgary, Calgary, AB T2N 1N4, Canada; zahra.bagheriashena@ucalgary.ca (Z.A.); liangs@ucalgary.ca (S.L.); 2 Department of Electrical and Software Engineering, University of Calgary, Calgary, AB T2N 1N4, Canada; yasaman.honarparvar@ucalgary.ca

**Keywords:** complex event detection, video processing, internet of things, cloud computing, spatial relationships detection, trajectory analysis, object tracking, object detection, computer vision

## Abstract

Complex event detection (CED) adds value to camera stream data in various applications such as workplace safety, task monitoring, security, and health. Recent CED frameworks have addressed the issues of limited spatiotemporal labels and costly training by decomposing the CED into low-level features, as well as spatial and temporal relationship extraction. However, these frameworks suffer from high resource costs, low scalability, and an increased number of false positives and false negatives. This paper proposes GICEDCAM, which distributes CED across edge, stateless, and stateful layers to improve scalability and reduce computation cost. Additionally, we introduce a Spatial Event Corrector component that leverages geospatial data analysis to minimize false negatives and false positives in spatial event detection. We evaluate GICEDCAM on 16 camera streams covering four complex events. Relative to a strong open-source baseline configured for our setting, GICEDCAM reduces end-to-end latency by 36% and total computational cost by 45%, with the advantage widening as objects per frame increase. Among corrector variants, Bayesian Network (BN) yields the lowest latency, Long Short-Term Memory (LSTM) achieves the highest accuracy, and trajectory analysis offers the best accuracy–latency trade-off for this architecture.

## 1. Introduction

Merriam-Webster defines an event as “the occurrence of something” [1]. Events can be categorized as either simple or complex. Simple events are atomic and independent, and cannot be decomposed into smaller events [2]. A complex event consists of a series of timestamped simple events that are logically or temporally interconnected [3]. While simple event detection is as straightforward as reading sensor measurements or alarms [4], complex event detection (CED) refers to the process of composing such timestamped simple events into meaningful higher-level events [3]. In video streams, a simple event is the presence of an object within a frame. When these objects are linked through spatial and temporal relationships, complex events can be formed [5]. In this paper, we define two mid-level events (Low-level events are simple events, and high-level events are complex events. Mid-level events are composed of low-level events and can form high-level events.). A spatial event occurs when two or more objects exhibit spatial relationships at the same time. A temporal event occurs when two or more spatial events exhibit temporal relationships. At the highest level, a complex event occurs when multiple temporal events co-occur or occur in sequence within a specific time window, conforming to a predefined pattern.

The rapid increase in video and camera content and advancements in Artificial Intelligence (AI) have led to a growing demand for automated video content analysis [6]. Effective CED enhances workplace safety, improves situational awareness, and enables automated monitoring of human activity [7], surveillance systems [8], healthcare [9], workers’ tasks monitoring [10], assembly operations monitoring [11], and contact tracing [12]. Many studies and frameworks have focused on improving CED accuracy, performance, cost efficiency, and scalability. Honarparvar et al. (2024) categorized CED methods into four main types [5]. This paper focuses on Object Detection and Spatiotemporal Matching (ODSM) methods. ODSM methods break CED into object detection within frames and the matching of event patterns based on spatiotemporal relationships. These patterns are typically queried from a complex event knowledge graph.

**Problem Statement:** Existing ODSM frameworks mostly rely on fully stateful computing architectures. In these frameworks, all relevant data for a complex event must be stored to enable querying against the knowledge graph. Maintaining these states across time windows is resource-intensive and increases the computational cost of CED. This becomes a critical issue when many objects are detected per frame or when a network of multiple camera streams is involved. This problem severely limits the scalability of CED. Another challenge is the low accuracy of spatial relationship matching. Traditional methods rely solely on bounding-box (bbox) topology to infer spatial relationships. This approach is highly sensitive to object detection errors, such as occlusions, low-quality frames, false positives and negatives, or perspective distortions. These issues propagate into incorrect spatial matches and ultimately result in incorrect complex event matching.

**Proposed approach**: GICEDCAM reorders the pipeline to address these issues: (i) a stateless, knowledge graph-driven gating and enrichment stage filters detections before they reach any stateful operator; (ii) a geospatial projection with mobility-aware routing standardizes geometry and directs moving versus fixed objects to specialized operators; and (iii) an online Spatial Event Corrector leverages short-horizon trajectories and fuzzy zone membership to impute likely missing spatial predicates prior to temporal composition. This placement and orchestration (rather than new detectors) underpins the scalability and robustness demonstrated in our evaluation.

Accordingly, the main contributions of this paper are summarized as follows:

- The design and implementation of a three-layer architecture that distributes CED workloads to reduce computational cost and increase scalability.
- The development of a Spatial Event Corrector component that predicts missing spatial events and reduces false positives and false negatives in relationship matching.

The proposed method is based on three main assumptions:

- We can access sufficient calibration data (e.g., camera calibration files or ground control points) to transform frame-based coordinates into geospatial coordinates.
- Camera locations and orientations are fixed throughout the CED process.
- Cameras submit frames to edge devices and the cloud.

In real-world deployments, the assumptions stated above may not always hold. First, calibration data may be incomplete or outdated due to hardware changes, camera relocation, or drift over time. This can be addressed through automated self-calibration algorithms, the use of natural scene features as reference points, or periodic maintenance checks. Second, maintaining stable bandwidth can be difficult in distributed camera networks, which may lead to delays or dropped events. Finally, environmental variability (e.g., changes in lighting or weather conditions) can further degrade detection performance. While this paper does not implement countermeasures for these conditions, prior work on adaptive and robust event detection offers potential solutions. For instance, Li et al. [13] discuss hierarchical feature learning to improve adaptability to varying contexts, Kang et al. [14] explore adaptive thresholding for resource-constrained video analytics, and MERN [15] demonstrates the benefits of multimodal data fusion to handle environmental challenges. Such approaches could be integrated in future extensions of GICEDCAM to improve robustness in operational deployments.

The remainder of this paper is organized into six sections. Section 2 reviews existing methods and frameworks for complex event detection. Section 3 describes the proposed GICEDCAM architecture and components. Section 4 presents the implementation details and experimental results and discusses the evaluation of outcomes. Section 5 concludes the paper and outlines future research directions.

## 2. Literature Review

Various methods have been proposed to detect complex events in videos, and numerous categorizations of CED approaches exist. Since this paper aims to improve complex event matching, we review existing work based on event-matching strategies, as categorized by Honarparvar et al. (2024) [5]. The first category includes Training and Predicting Videos (TPV) approaches. TPV approaches are based on supervised or semi-supervised learning models trained on labelled video datasets to predict complex events. The major challenges of these methods are the limited training data [16] and noisy labels [17]. TPV methods typically require large-scale, high-quality labelled datasets, which are expensive and time-consuming to produce. The second category is Zero-Example Prediction (ZEP). ZEP frameworks use semantic search algorithms to match user-generated queries with video corpus metadata [18]. These search algorithms reduce the cost of labelling and training. However, they depend heavily on the availability of textual metadata, which restricts their applicability to web-based videos. The third category, known as Multi-Source Fusion (MSF), incorporates auxiliary data sources (e.g., audio, motion sensors) to improve event detection accuracy [19]. Despite their benefits, MSF methods are constrained by their dependency on the quality of external data and often suffer from processing latency [20].

Another category of event matching methods is called ODSM-based. ODSM approaches do not require spatiotemporal labelling. These approaches rely on lightweight models for object detection, followed by pattern-matching techniques to identify spatial and temporal relationships. Unlike other methods, ODSM operates solely on video stream data and does not require textual information or external sources. Understanding object interactions and spatiotemporal relationships plays a central role in aligning detected events with predefined patterns in CED. Techniques such as entity-centric feature pooling focus on object-human interactions by extracting localized spatiotemporal relationships [21]. EventNet was among the earliest ODSM frameworks, which introduced a video ontology framework to link object relationships with complex event concepts and enable semantic querying within video content [22]. Other innovations include trajectory-based models, where event semantics are assigned through hypergraph pairing [23,24], and frameworks for abnormal human behavior detection via trajectory clustering using dense descriptors [25]. Hierarchical models have also been developed to reduce error propagation in CED by creating layered relationships from frame-level features to the temporal activity concept [13].

As querying performance became a bottleneck, several frameworks emerged to improve efficiency. VideoStorm addressed resource allocation with lag- and quality-aware querying [26], while BLAZEIT introduced FrameQL to reduce DNN inference costs in video analytics [14]. Hybrid workflows (e.g., Khan et al.) leveraged logical reasoning over simple event detection to identify complex patterns [27]. VIDCEP represents a major advancement by combining a flexible Video Event Query Language (VEQL) with a Video Event Knowledge Graph (VEKG) to enable multistep spatiotemporal event matching [15,28,29]. Subsequent models like MERN, TAG, and NLP-guided ontologies enhanced semantic representation and reduced processing complexity [30,31,32]. The Notification-Oriented Paradigm (NOP) recently proposed an efficient chain-based querying mechanism, although it remains constrained to specific event types [33]. Collectively, these developments underscore a growing emphasis on semantic-rich modelling and computational efficiency in complex event-matching frameworks.

Despite these advancements, ODSM-based frameworks still face critical limitations. Frameworks like VIDCEP, BLAZEIT, and NOP are often not cost-efficient. Given the high video streaming rate (e.g., 30 fps), storing and maintaining frame-level data in memory for extended periods leads to significant computational overhead. Moreover, these frameworks typically do not offer generalized solutions to reduce spatial and temporal matching errors caused by object occlusion or missed detections. As a result, they remain prone to false negatives and incur high processing costs. Although ODSM frameworks reduce reliance on labels and improve querying mechanisms, challenges remain regarding cost efficiency, scalability, and robustness to detection errors. In the next section, we propose a novel framework to address these issues by distributing CED workloads across computational layers and introducing error-correcting components. Table 1 summarizes the reviewed ODSM-based CED frameworks’ strengths and limitations.

## 3. Method Overview

This section introduces the methodology of the proposed GICEDCAM framework for improving CED in camera streams. It is organized into three main subsections. Section 3.1 presents the architecture of the GICEDCAM data pipeline and the structure of the proposed complex event knowledge graph. Section 3.2 details the Spatial Event Corrector component, including three approaches for predicting missing spatial events. Section 3.3 describes the Real-Time Trajectory Corrector, which includes the tracking re-identifier and trajectory spatial enhancer.

### 3.1. GICEDCAM Framework Design

**Problem recap** (see Section 1). Prior stateful pipelines inflate memory/latency, and bbox-only spatial matching propagates detector errors; we address these with the following innovations:

- **Enhancing the Complex Event Knowledge Graph**: The complex event knowledge graph (KG) is revised to incorporate additional geospatial functions and entities. This enhancement is crucial for effectively addressing the challenges posed by false negatives and false positives associated with spatial events.
- **Improving Computational Resource Allocation**: The computational burden of low-level feature extraction is relocated to the edge computing components. This strategic shift will allow cloud computing components to concentrate on the more intricate tasks of spatial events, temporal events, and complex events matching.
- **Stateless Spatial Matching**: The entire process of spatial event matching is transitioned to stateless data processing components. In contrast, the temporal and complex event matching will remain within the purview of stateful matching components.

We next describe the GICEDCAM knowledge graph (Section 3.1.1) and data pipeline (Section 3.1.2).

#### 3.1.1. GICEDCAM Knowledge Graph

KGs are foundational to every CED framework, as they define the semantic structure of events and the relationships among involved entities. Designing an effective KG requires addressing criteria such as event pattern matching efficiency, scalability, and the ability to support complex spatial and temporal relationships [31]. In the context of GICEDCAM, we design a KG that meets these criteria while aligning with the framework’s multi-layered data pipeline and processing flow.

**Figure 1:**
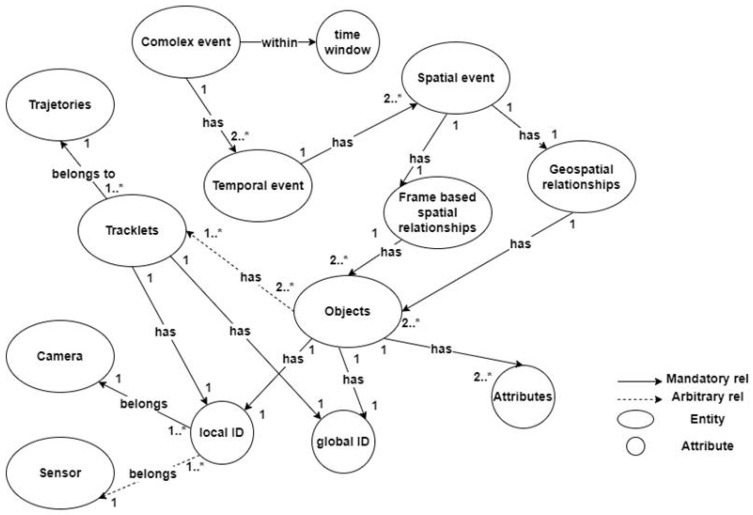
GICEDCAM knowledge graph.

As shown in Figure 1, the KG schema includes essential entities, attributes, and relationships. Based on the schema, at the lowest level of event features, every object must have at least two attributes (i.e., geospatial zone and mobility status). The geospatial zone is necessary to address the missing spatial events issue. The mobility status attribute helps reduce computational costs as we no longer need to run complex processes for fixed objects. There could be more attributes per object, such as color histogram, object speed, and other low-level features, which are stored in the attribute vector. Each object must have a local ID that is generated by an IoT sensor or a camera. Objects must also have a global ID, which identifies the object in the real world and is the result of the matching between various local IDs. Objects include at least one tracklet (A tracklet is a temporal sequence formed by linking consecutive detections of a single object across video frames, which share the same tracking ID.), if its mobility status is the “moving object”. Each tracklet is a part of one trajectory, and it also has a local ID (which is generated by the camera tracking algorithm) or a global ID (which identifies the tracklet ID in all joined trajectories of the object). Tracklets and trajectories are important parts of the KG since moving objects are mandatory entities of any complex event. In short: “no movement, no event.”

Each spatial event has one instance of geospatial and frame-based spatial relationships. The frame-based spatial relationships are defined as topological relationships of multiple objects’ bbox in a frame. Therefore, every frame-based spatial relationship involves at least two objects. Topological relationships in videos can be encoded using the Egenhofer matrix for 2D objects [34]. To avoid false positives and false negatives in detecting spatial events, we need to discover spatial relationships in a geospatial coordinate system. For example, a person’s hand bbox intersects a door handle while standing away from the door. We can avoid this false positive error by determining geospatial relationships between objects. The geospatial relationships are directional (e.g., west of), distance-based (e.g., nearby), or contextual (e.g., objects within the same region).

Temporal events can be encoded in 13 time intervals, including before, meets, overlaps, during, starts, finishes, equals, is met by, is overlapped by, contains, is started by, is finished by, and after. For example, the “*person picks up the bottle*” spatial event time window overlaps the “*bottle is on the table*” spatial event, which forms the “*person picks the bottle from the table*” temporal event.

The following is the entity set (nodes) of the proposed KG:

- **Camera**: {camera_id, name, H (3 × 3), ts_calib, crs: cartesian-m}
- **Zone**: {zone_id, polygon, version, crs, bbox, semantics}
- **Object**: {global_id, class, attrs, ts_first, ts_last}
- **LocalObject (per sensor)**: {local_id, camera_id, class}
- **Tracklet**: {tracklet_id, camera_id, local_id, ts_start, ts_end}
- **Trajectory**: {traj_id, global_id, ts_start, ts_end}
- **Observation (immutable)**: {obs_id, ts, u, v, x, y, conf, bbox}
- **SpatialEvent**: {se_id, type, ts, corrected:boolean, conf}
- **TemporalEvent**: {te_id, type, t_start, t_end}
- **ComplexEvent**: {ce_id, type, t_start, t_end, query_id}

The following is the relationships set (edges) of the KG:

- **(:LocalObject)-[:SAME_AS]->(:Object)** (many→1)
- **(:Object)-[:HAS_TRACKLET]->(:Tracklet)** (1→many)
- **(:Tracklet)-[:PART_OF_TRAJ]->(:Trajectory)** (many→1)
- **(:Observation)-[:OF]->(:LocalObject)** (many→1)
- **(:Observation)-[:PROJECTED_BY {camera_id}]->(:Camera)**
- **(:Observation)-[:IN_ZONE {ts, μ}]->(:Zone)**
- **(:SpatialEvent)-[:ABOUT]->(:Object)** (many→1)
- **(:TemporalEvent)-[:COMPOSES]->(:SpatialEvent)** (many→many)
- **(:ComplexEvent)-[:COMPOSES]->(:TemporalEvent)** (many→many)

The following includes the required integrity constraints and the indexes script to build the proposed KG (Box 1):

Box 1. **KG integrity constraints and indexes.**

// Uniqueness

**CREATE CONSTRAINT** obj_gid **IF NOT EXISTS**

**FOR** (o:Object) **REQUIRE** o.global_id **IS UNIQUE**;

**CREATE CONSTRAINT** loc_lid **IF NOT EXISTS**

**FOR** (l:LocalObject) **REQUIRE** (l.camera_id, l.local_id) **IS UNIQUE**;

**CREATE CONSTRAINT** trk_id**  IF NOT EXISTS**

**FOR** (t:Tracklet)    **REQUIRE** t.tracklet_id **IS UNIQUE**;

**CREATE CONSTRAINT** traj_id **IF NOT EXISTS**

**FOR** (r:Trajectory)  **REQUIRE** r.traj_id **IS UNIQUE**;

**CREATE CONSTRAINT** zone_id **IF NOT EXISTS**

**FOR** (z:Zone)  **REQUIRE** (z.zone_id, z.version) **IS UNIQUE**;

**CREATE CONSTRAINT** se_id**   IF NOT EXISTS**

**FOR** (s:SpatialEvent)  **REQUIRE** s.se_id **IS UNIQUE**;

**CREATE CONSTRAINT** te_id **IF NOT EXISTS**

**FOR** (t:TemporalEvent) **REQUIRE** t.te_id **IS UNIQUE**;

 

// Query-speed indexes

**CREATE INDEX** obs_ts **IF NOT EXISTS**

**FOR** (o:Observation)  **ON** (o.ts);

**CREATE INDEX** se_type_ts **IF NOT EXISTS**

**FOR** (s:SpatialEvent)  **ON** (s.type, s.ts);

**CREATE INDEX** te_type_t **IF NOT EXISTS**

**FOR** (t:TemporalEvent)  **ON** (t.type, t.t_start);

**CREATE INDEX** obj_class **IF NOT EXISTS**

**FOR** (o:Object)    **ON** (o.class, o.ts_last);

The proposed KG is scalable because the hierarchical layering (complex → temporal → spatial) enables efficient graph partitioning, indexing, and parallel traversal. Hierarchical layering also allows for progressive and modular event querying and processing. Furthermore, a clear distinction between geospatial and frame-based relationships supports hybrid spatial queries. Finally, the proposed KG accommodates multi-entity and multi-layered complex relationships by breaking down events into low-level, mid-level, and high-level events.

#### 3.1.2. GICEDCAM Data Pipeline

**Figure 2:**
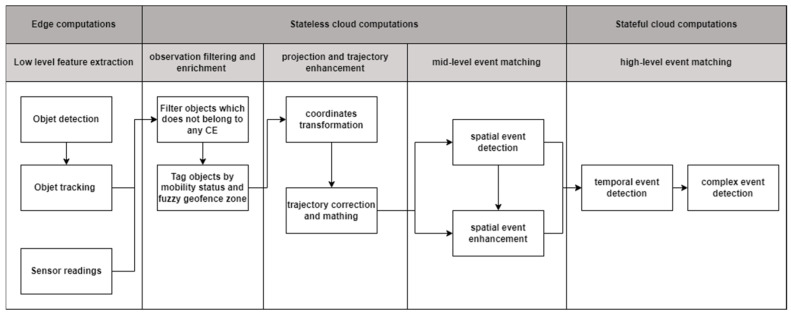
GICEDCAM framework data pipeline.

The GICEDCAM data pipeline performs data ingestion, transformation, and event detection. The main criteria for designing the GICEDCAM data pipeline are to increase scalability, reduce cost, and increase the accuracy of processing and detecting complex events in near real-time compared to traditional CED frameworks like VIDCEP [15]. The GICEDCAM framework is designed as shown in Figure 2.

The GICEDCAM data pipeline comprises three computation layers: edge, stateless, and stateful. In the edge computation layer, low-level feature extraction is handled using edge computations. Low-level feature extraction converts raw frames and sensor readings into compact features. In the first task, objects are detected using a Convolutional Neural Network (CNN) for object detection. Honarparvar et al. demonstrated that YOLO is a suitable CNN architecture for detecting simple events or objects in videos [35]. The next step is to track objects and identify them as unique objects in the camera streams. The DeepSORT algorithm has been widely used for tracking multiple objects in one video stream and is well-aligned with CNN object detection, such as YOLO [36]. Other sensors (e.g., Inertial Measurement Unit (IMU), voice recorder, or temperature sensor) may also publish low-level features of the objects. The extracted low-level features should include time, location, and observation type as mandatory fields. Moving low-level feature extraction to the edge computation layer reduces latency and bandwidth since this layer publishes small-sized constructed data instead of large-sized camera streams. Moreover, the scalability is increased since edge devices easily scale linearly, and the computational costs are distributed to edge devices.

The stateless computation layer runs the processes that do not need the reserved state. This layer consists of three sublayers. In the first sublayer, observations are filtered and enriched with mobility status and geospatial zones. Filtering objects against the KG database reduces the number of rows for CED and increases computational efficiency. The filtering component queries the objects’ type in the KG database and returns all complex-event patterns that involve any of the detected object types. The sample Cypher query to find registered objects is as follows (Box 2):

Box 2. **The sample Cypher query to find registered objects.**

**MATCH**(ce:ComplexEvent)-[:**HAS_TEMPORAL**]->(te:TemporalEvent)-[:**HAS_SPATIAL**]->(se:SpatialEvent)-[:**INVOLVES**]->(ob:Object)

**WHERE** op.label = $label

**RETURN TRUE**

The next component enriches the objects by the mobility status by querying the objects’ type from the KG database. The mobility status is either “fixed” or “moving”. Assigning the mobility status helps process fixed or moving objects in different ways. For example, a fixed object’s location could be simply an average of all recorded frames’ locations. However, moving objects are required to be processed by more complicated algorithms (see Section 3.2.3). Objects are also labelled based on the geospatial zones where they are located. The zone assignment would help reduce false negatives of spatial event detection (see Section 3.2.3).

The next sublayer performs projection and trajectory enhancement. This component retrieves the enriched objects and projects them onto a geospatial plane. Some well-known approaches, such as homography-based transformation [37] or camera calibration techniques [38] transform the location of the detected object in the frame coordinate system to a geospatial coordinate system like the Universal Transverse Mercator (UTM). We provided the source code and samples of efficient coordinate transformations in the CameraObjectMapper v1.0 (https://github.com/sepehr89/CameraObjectMapper accessed on 1 March 2025) GitHub repository. For each detection of object *o* at time *t*, we select an image point (*U_t_*^(*o*)^, *V_t_*^(*o*)^) (center of the object’s bbox) and map it to world coordinates via the static camera homography *H_c_* ∈ R^3×3^ (one per camera). Equation (1) shows how to project the image coordinates into a geospatial planar coordinate system. The *x*_1_ and *x*_2_ are the unnormalized ground-plane coordinates, and *x*_3_ is the projective scale.

(1) $$\tilde{x}={H_{c}[u_{t},v_{t},1]}^{T},\;x_{o}(t)=[\frac{\tilde{x_{1}}}{\tilde{x_{3}}},\frac{\tilde{x_{2}}}{\tilde{x_{3}}}]$$

The projected coordinates are combined with other location data taken from IMU, Global Positioning System (GPS), or Wi-Fi to reduce the false positives in spatial-event detection. For example, a person’s hand intersects with a door handle while he/she is far from the door. The component returns the person’s geospatial coordinates to determine proximity to the door handle. The next component of this sublayer enhances the trajectory data in terms of tracking identification accuracy and trajectory geometry. This component fixes the gaps in moving objects’ trajectories and reduces false negatives in temporal event detection. More details are explained in Section 3.2.3.

The mid-level event-matching sublayer handles spatial event matching and spatial event enhancement components. Both of these components are handled by stateless computations because spatial event matching considers objects’ spatial relationships at a specific point in time and does not require storing the state. Spatial event detection matches the detected spatial relationships with patterns of frame-based spatial relationships and geospatial relationships in the KG database. The following Cypher query is an example of how we can detect a spatial event (Box 3).

Box 3. **Cypher query for spatial event detection.**

**MATCH** (se:SpatialEvent)-[:**INVOLVES**]->(o1:Object),

   (se)-[:**INVOLVES**]->(o2:Object),

   (se)-[:**USES_RELATIONSHIP**]->(r:SpatialRule)

**WHERE** o1.label = ‘person’ **AND** o2.label = ‘car’ **AND** r.type = ‘near’

**RETURN** se

However, spatial events may be overlooked due to various factors, including frame quality issues, false negatives in object detection, or occlusion phenomena. The primary objective of spatial event enhancement is to minimize the likelihood of false negatives by employing knowledge-based, data-driven, or trajectory-focused approaches. Section 3.2 provides more details on how GICEDCAM identifies the gaps in spatial events. This stateless sublayer promotes scalability. The atomic processes are handled by one-time processing functions, which use resources more efficiently. These functions are triggered only when needed and terminate upon completion. Such a design significantly reduces processing costs. Additionally, these stateless computations enhance fault tolerance due to the modular nature of the computational units, as well as the loosely coupled microservices and components they comprise.

In the last layer, we leverage stateful computations to identify temporal relationships among spatial events and detect complex events. This functionality cannot be adequately managed through stateless processing, as temporal events are inherently not isolated or atomic; they encompass various factors such as pattern accumulation, the sequence of patterns, time windows, and the continuity of objects over time. In this layer, we account for event windows to detect patterns within fixed temporal intervals. We implemented session tracking to maintain the spatial events state for the defined event time window. Additionally, we utilize pattern memory to accumulate partial matches, which contribute towards the identification of full complex events. The sample Cypher query to match temporal and complex events with the KG patterns database is presented as follows (Box 4):

Box 4. **Cypher query to match temporal and complex events with the KG patterns database.**

**MATCH**(ce:ComplexEvent)-[:**HAS_TEMPORAL**]->(te:TemporalEvent)

   -[:**HAS_SPATIAL**]->(se:SpatialEventPattern)

**RETURN** ce, te, se

Algorithm 1 provides more details on how the stateful event matching works. The stateful matcher in GICEDCAM operates over a stream of spatial events, a query window *W* (maximum allowed duration for a complex event), and a watermark *ω* (maximum event time observed minus allowed lateness). Each spatial event is represented as *e* = ⟨type, bindings, *t*, conf, corrected⟩, where *type* is the spatial event predicate, *bindings* is the map from pattern variables to KG identifiers, *t* is the event time of the spatial event, *conf* is the event confidence, and *corrected* is a Boolean flag (true if emitted by the Spatial Event Corrector). The query pattern is compiled into a finite-state machine; the engine maintains partial matches *M*[*q*] keyed by automaton state *q* and current bindings, each storing {{bindings, start_time, last_time, (optional) confidence}}. Upon receipt of event *e*, the engine advances any partial whose next transition is enabled (i.e., the label matches *e*.type, the event’s bindings unify with the partial’s bindings without conflict, the required Allen-interval guard and gap constraints hold relative to last_time, and the resulting span (*t* − start_time) ≤ *W* (optionally conf ≥ *θ*)). If the pattern can start with *e*, a new partial is seeded with start_time = last_time = *t*. A partial that reaches an accepting state is emitted as a complex event only when (last_time − start_time) ≤ *W* and last_time ≤ *ω*, ensuring finality under out-of-order and corrected inputs. Corrected events are additive only; if their event time *t* ≤ *ω* on arrival, they are too late. This makes the event matching robust to missing events since the corrector is additive; when imputed events arrive before *ω*, they can complete/extend matches without mutating prior facts. Finally, any partial with (now − start_time) > *W* is pruned to bound memory and enforce the semantic duration. The pruning stage helps release the memory and reduces computational cost.
**Algorithm 1.** Stateful Complex Event Matching in GICEDCAM.**Input**: stream of spatial events e = ⟨type, bindings, t, conf, corrected⟩   window W, watermark ω **State**: partial matches M[q] keyed by (q, bindings) upon spatial event e at time t:  // events are pre-filtered & projected // 1) advance existing partials **for each** (q, b) **in** M **where** δ(q, e.type, b, t) **is enabled**   q′ ← δ(q, e.type, b, t)  // checks Allen relation & gap constraints   b′ ← unify(b, e.bindings)  // variable binding; reject on conflict   **upsert** M[q′] **with** {bindings = b′, start_time(b), last_time = t} // 2) possibly start a new partial **if** δ(q0, e.type, e.bindings, t) **enabled**   **upsert** M[q1] **with** {bindings = e.bindings, start_time = t, last_time = t} // 3) emit completes when safe **for each** partial **in** M **with** state q **∈** F   **if** (last_time − start_time) ≤ W **and** last_time ≤ ω     **emit** ComplexEvent(partial.bindings, [start_time, last_time])     **delete** partial // 4) prune stale partials (semantic bound) delete any partial with (current_time − start_time) > W

### 3.2. Spatial Event Corrector

The Spatial Event Corrector is triggered when the system identifies a potential gap in spatial event detection based on KG-defined patterns. The Spatial Event Corrector runs in parallel with spatial-event detection. It estimates the probability of a missing event using contextual cues and past observations. For instance, consider a scenario where a person is observed at time (t_0_) and a bottle positioned at an intersection with a table is noted at time (t_1_). Subsequently, at time (t_2_), the individual is seen standing near the table, apparently preparing to “*pick up the bottle*”, but this action goes undetected due to the occlusion of the bottle by the person’s body. By the time (t_3_), the object detector successfully identifies the person holding the bottle. Further, the system detects the individual opening the door and exiting the room at time (t_4_). The observation that the person holds the bottle after passing the table indicates that the bottle was picked up earlier.

In this context, the GICEDCAM framework employs the Spatial Event Corrector component to initially identify potential gaps in spatial event detection and subsequently predict these missing spatial events with a calculated level of confidence, utilizing contextual clues and the knowledge graph. The Spatial Event Corrector in GICEDCAM evaluates three representative approaches, including Bayesian Networks (BN), Long Short-Term Memory (LSTM), and trajectory analysis, which are chosen for their complementary strengths in addressing uncertainty, temporal dependencies, and spatial continuity. BN was selected for its capability to explicitly represent probabilistic dependencies among spatial events and perform inference with incomplete or noisy observations, as demonstrated in hierarchical reasoning approaches such as Li et al. [23]. LSTM was included due to its proven capacity to capture long-range temporal dynamics in sequential event data, consistent with the temporal modelling advantages highlighted in Kang et al. [25]. The trajectory analysis method was chosen for its computational efficiency and interpretability, leveraging geometric movement constraints to correct missing events, in line with object movement modelling seen in MERN [27]. While alternative methods such as Graph Neural Networks (GNN) can model complex relational structures in spatiotemporal graphs, our selection prioritized methods with lower training data requirements, established interpretability, and reduced computational overhead; these are all key considerations for real-time, resource-constrained deployments. Future extensions of GICEDCAM may explore GNN-based correctors when computational budgets and labelled training data are sufficient. In this section, we explain the approaches in detail, and in Section 4.3, we will compare them in terms of accuracy and latency.

Algorithm 2 explains more details on the Spatial Event Corrector component. The corrector operates on active expectations *E*_*S*_ = ⟨*S*, bindings, [*t*_*a*_,*t*_*b*_]⟩ derived from the query pattern (anchors define when a spatial predicate *S* should occur), an observation buffer *O*_*K*_ containing the last *K* seconds of projected positions *x*(*t*), fuzzy zone memberships *μ*_*Z*_(*x*) class labels, and frame-topology flags, and a configuration that selects exactly one method ∈ {BN, LSTM, TRAJ} together with thresholds (*τ*_BN_, *τ*_LSTM_, *τ*_traj_), a small probe delay Δprobe (patience before correction), de-duplication (dedup) tolerance *ε*_*t*_, padding δ, and a wall-clock budget B ms. Method-specific parameters are: BN structure/CPDs ΘBN; LSTM model weights *M*_LSTM_ (plus window length/stride); and trajectory assets (ROI maps *μ*_ROI_, historical exemplars *H*, FastDTW radius *r*, blend *β*). For each *E*_*S*_, the component performs gap detection: if the current time is ≥*t*_*b*_+Δ_probe_ and no raw instance of *S* with the given bindings exists in [*t*_*a*_, *t*_*b*_], it proceeds; otherwise, it returns. It then assembles evidence by slicing *O*_*K*_ over [*t*_*a*_−*δ*, *t*_*b*_+*δ*]. In BN mode, it runs exact inference to obtain *p*_*t*_ = P_r_[*S* = 1∣*E*_*t*_] and selects t* = argmax*t* ∈ [*t*_*a*_,*t*_*b*_]*p*_*t*_; if *p*_t_* ≥ *τ*_BN_, it emits with conf = *p*_t_*. In LSTM mode, it evaluates the sequence with weights *M*_LSTM_ over sliding windows on [*t*_*a*_, *t*_*b*_], chooses t* = argmax *p*_*t*_, and emits if *p*_t_* ≥ *τ*_LSTM_. In trajectory mode, it partitions the trajectory by MDL, scores candidate segments via a blend *β*_FastDTW_sim_ + (1−*β*)*μ*^−^_ROI_, and, if the best score ≥ *τ*_traj_, sets t* = argmax_*p*∈*σ*_ *μ*_ROI_(*p*) with conf = score. Before emission, the corrector de-duplicates against raw observations: if a raw *S* exists within *ε*_*t*_ of t*, it suppresses the correction. Otherwise, it adds (never mutates) a new spatial-event record ⟨type = *S*, bindings, t*, conf, corrected = true⟩. Finality and eligibility are enforced downstream by the stateful matcher (watermark *ω* and window *W*); the corrector itself remains stateless and respects the budget *B*. More details on LSTM, BN, and trajectory-based methods are explained in Section 3.2.1, Section 3.2.2 and Section 3.2.3.
**Algorithm 2.** Spatial Event Corrector.**Inputs**: Active expectations E_S = ⟨S, bindings, [t_a, t_b]⟩ // from pattern context & anchors Observation buffer  O_K    // last K s of {x(t), μ_Z(x), class, bbox flags} Config        method ∈ {BN, LSTM, TRAJ}, ∆_probe, ε_t, δ, B Thresholds      τ_BN, τ_LSTM, τ_TRAJ BN params      Θ_BN LSTM params     M_LSTM (model weights), T (window), stride Trajectory params  μ_ROI(·), exemplars H, FastDTW radius r, blend β **State**: none (stateless; queries only) **procedure** CORRECTOR(E_S): // ----- GAP DETECTION ----- **let** ⟨S, bindings, [t_a, t_b]⟩ = E_S **if** now < t_b + Δ_probe: **return**      // wait a small patience window **if exists** RAW SpatialEvent(type = S, bindings, t_raw **∈** [t_a, t_b]) in O_K:    **return**              // no gap → nothing to correct // ----- EVIDENCE ASSEMBLY ----- E ← slice O_K for variables in ‘bindings’ over [t_a − δ, t_b + δ] **if** E is **empty**: **return** // ----- SINGLE-METHOD IMPUTATION (chosen by config) ----- **switch** method:  **case** BN:   …   **if** p < τ_BN: **return**   conf ← p  **case** LSTM:   …   **if** p_t(t*) < τ_LSTM: **return**   **conf** ← p_t(t*)  **case** TRAJ:   …   **if** best = Ø **or** best.score < τ_TRAJ: **return**   t* ← **argmax**_{p∈best.σ} μ_ROI(p)            // most plausible time inside segment   **conf** ← best.score // ----- DEDUP AGAINST RAW EVENTS ----- **if exists** RAW SpatialEvent(type = S, bindings, t_raw) **with** |t_raw − t*| ≤ ε_t in O_K:   **return**                         // prefer raw observation // ----- EMIT ADDITIVE CORRECTED EVENT ----- **emit** SpatialEvent ⟨type = S, bindings, t* = t*, conf = conf, corrected = **true**⟩ // ----- TIME BUDGET GUARD (optional) ----- **ensure wall-clock** time ≤ B ms (degrade by skipping LSTM or lowering DTW radius if needed)

#### 3.2.1. Bayesian Networks

Bayesian Networks (BNs) are widely used for event prediction and predictive analytics. [39]. They are particularly effective in managing missing data, seamlessly integrating domain knowledge [40], and demonstrating high efficacy with small sample sizes [41]. Additionally, BNs are beneficial in preventing overfitting, making them versatile for various predictive tasks [42]. A BN is a probabilistic graphical model that represents a set of variables and their conditional dependencies using a directed acyclic graph (DAG). In this network, each node corresponds to a random variable, while the edges signify the probabilistic dependencies that exist between these variables. The network is characterized by Conditional Probability Distributions (CPDs), which quantitatively describe how the probability of a specific variable is influenced by its parent nodes.

BNs are useful in CED, particularly when some events are missing or uncertain. They facilitate probabilistic reasoning, allowing for inference even when only partial data is accessible. The foundation of a BN lies in Bayes’ theorem, which is succinctly encapsulated in Equation (2). This equation estimates the probability of the evidence *E*, under the condition *C,* while *H* is considered our hypothesis. More details of the BN approach are explained in Appendix A.1.

(2) $$P\left(E|C\right)=\sum P\left(E|H_{i},C\right).P(H_{i}|C)$$

This approach incurs little latency and computational overhead, but its effectiveness heavily relies on the probability values assigned by experts to each node in the BN. These assigned values influence the accuracy of detecting missing events. Therefore, in this paper, we use a dynamic probability estimation method to update CPD values in real-time. Specifically, we define a time window (e.g., 8 h) during which we calculate the likelihood of observing specific CPD values for the BN nodes (e.g., “*person near the table*”) at regular intervals. For the initial time window, we utilize the probability values determined by experts.

#### 3.2.2. Long Short-Term Memory

LSTM networks, a specialized type of Recurrent Neural Networks (RNNs), are particularly adept at modelling sequential dependencies within time-series data [43]. When some events are occluded or not explicitly observed in a dataset, LSTM can leverage past and future contexts to infer their occurrence [44]. Traditional probabilistic approaches, such as BN, require predefined CPDs, but LSTMs dynamically learn patterns from training data, rendering them particularly effective for estimating missing events in real-world applications, such as video-based event detection. Furthermore, while BNs struggle to capture the temporal dependencies inherent in event sequences, LSTMs excel at identifying and learning time-series patterns [45].

LSTMs operate by maintaining a memory state that selectively retains relevant information from past time steps using gates (input, forget, and output gates). When an event is missing, the network reconstructs the missing pattern based on previously observed movement sequences. For example, in a human-object interaction scenario, if a “*bottle pickup*” event is occluded, but the person’s trajectory suggests movement near a table followed by the bottle appearing in their possession, the LSTM can learn this transition from historical data and predict the missing event with a high degree of confidence. This capability arises from the LSTM’s ability to capture spatiotemporal dependencies, allowing it to infer events based on temporal patterns rather than relying solely on instantaneous observations. More details of LSTM approach is elaborated in Appendix A.2.

LSTMs depend heavily on data quality and can achieve high accuracy if the prediction model is well-trained. However, the requirement for labelled data makes this approach less cost-effective. Furthermore, in the existing stateless architecture, LSTM-trained models need to be loaded each time the Spatial Event Corrector is triggered. This repeated loading consumes significant memory resources and contributes to increased latency.

#### 3.2.3. Trajectory Analysis

Complex events in videos involve moving and fixed objects. In general, a complex event cannot occur unless at least one object changes its location across consecutive frames. Therefore, tracking objects’ locations and understanding objects’ trajectory patterns can play a significant role in detecting complex events. When dynamic trajectory patterns are correctly identified, missing spatial or temporal events can also be inferred more reliably.

Trajectories provide valuable information about spatiotemporal patterns of moving objects [46]. We can partition and cluster trajectories to see where and when moving objects follow unique patterns [47]. Subsequently, these identified patterns can be integrated with semantic data to infer potential activities occurring at specific Points of Interest (POIs) or Regions of Interest (ROIs) [48]. A variety of methods have been developed that rely solely on trajectory data for activity recognition, including neural networks [49], Hierarchical Hidden Markov Model (HHMM) [50], Principal Component Analysis (PCA) [51], and Latent Dirichlet Allocation (LDA) [52]. However, these methods require manual labelling or the assignment of probabilistic transition values. Moreover, these methods often involve complex computations that demand considerable computational resources. On the other hand, there is an opportunity to enhance analysis by leveraging complementary information, such as the locations of static objects and relevant spatial events.

**Figure 3:**
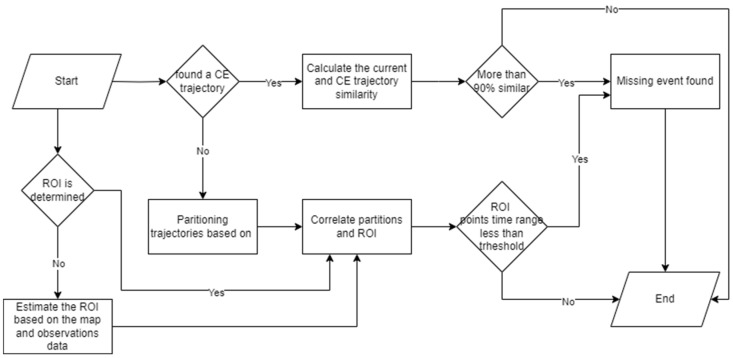
Workflow of the trajectory analysis approach to detect a missing event.

Figure 3 presents a detailed workflow for identifying missing events through trajectory data analysis. The first step is to query historical trajectory datasets which are related to the target complex event and the potential spatial event. If a trajectory is found, we run the trajectory similarity function to calculate the similarity between the current trajectory and the found historical trajectory. We can claim that two objects did similar activities if their trajectories are similar enough [53]. Hence, we conclude that the missing spatial event occurred at the corresponding location on the established complex event’s trajectory.

Among trajectory similarity calculation algorithms, Dynamic Time Warping (DTW) stands out for its high accuracy in measuring temporal similarities [54,55]. However, DWT suffers from high complexity *O*(*n*^2^) due to the nonlinear calculation nature [56]. To address this limitation, FastDTW has been developed as a linear approximation of DTW, which significantly enhances the computational speed when analyzing large datasets. [57]. Unlike the traditional DTW method, which necessitates the computation of a full *O*(*n*^2^) distance matrix, FastDTW only refines certain regions, thereby accelerating the overall computation process. The FastDTW algorithm operates by reducing the trajectory size to $n/2^{k}$ at each level k, executing the standard DTW on a coarser resolution. Then it applies DTW at each level, resulting in complexity that can vary between *O*(*n*) to *O*(*nlog*(*n*)). After applying FastDTW calculations, the similarity is calculated based on Equation (3), and if it exceeds the threshold of 90%, the spatial event location from the similar trajectory is adopted and subsequently reported as the missing event for the current trajectory.

(3) $$Similarity(T_{1},T_{2})=1-\frac{FastDWT(T_{1},T_{2})}{max(length(T_{1}),length(T_{2}))}$$

**Figure 4:**
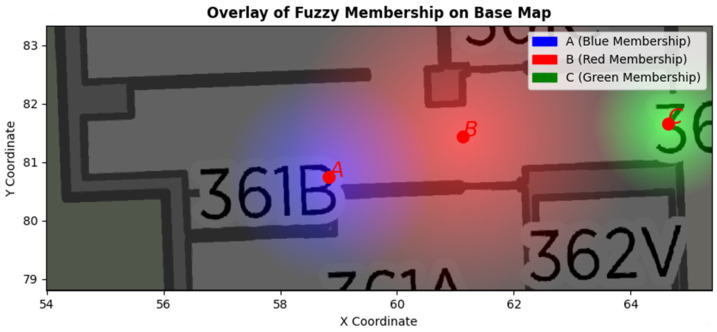
Fuzzy ROI map of the person picking the bottle, the person holding the bottle and moving to the door, and the person opening the door.

Meanwhile, we check whether we previously extracted ROIs from the datasets. In this context, ROIs represent potential areas where spatial events may occur. For example, a buffered zone around a table could serve as a probable location for the “*picking up a bottle*” spatial event. ROIs can be manually determined by defining the geometry and location of the region of interest. Alternatively, they may be estimated based on observational data, such as the detected location of a table, along with a buffer zone surrounding it. These ROIs can overlap since some regions have the potential for more than one spatial event occurrence. For example, certain regions have a high likelihood of both “person holds bottle” and “near table” events. Therefore, ROIs are conceptualized as fuzzy regions based on the probability of event occurrence. Figure 4 illustrates an example of fuzzy ROIs in an area. There are three ROIs. ROI_A_ identifies the ROI for a person who picks up the bottle on the table. The closer the person is to location A, the more membership value he gets for ROI_A_. The ROI_B_ is the region where the person holds the bottle and moves towards the door. The ROI_C_ represents the area where it is most likely for a person to pause and open the door. Fuzzy membership functions can be formulated uniquely for each ROI. For example, the membership value of ROI_A_ could be quantified in Equation (4), where *R* is the maximum radius of ROI from point A.

(4) $$M(x,y)=\left\{\begin{array}{c} 1-\frac{\sqrt{{\left(x-x_{A}\right)}^{2}+{\left(y-y_{A}\right)}^{2}}}{R}\;\;\;\;\;\;\;if\;y\geq y_{c}\;and\;\sqrt{{\left(x-x_{A}\right)}^{2}+{\left(y-y_{A}\right)}^{2}}\leq R\\ 0\;\;\;\;\;\;\;\;\;\;\;\;\;\;\;\;\;\;\;\;\;\;\;\;\;\;\;\;\;\;\;\;\;\;\;\;\;\;\;\;\;\;\;\;\;\;\;\;\;\;\;\;\;\;\;\;\;\;\;\;\;\;\;\;\;\;\;\;\;\;Otherwise \end{array}\right.$$

These ROIs intersect with the partitioned trajectories. The points within the trajectory segment that intersect these ROIs are identified and assigned membership values based on the corresponding fuzzy function for each ROI. Then the detection time range of all intersected points is calculated, and if they fall within the allowed time range, the points are selected as the missing events. The confidence level of the missing event is reported as the average of the fuzzy membership values of all intersected points.

Trajectory partitioning is another essential component of detecting missing events. The objective of this component is to divide the trajectory points into non-overlapping segments that have different movement patterns. Mashud et al. proposed a partitioning algorithm based on the Minimum Description Length (MDL) with the complexity of *O*(*N*) [58]. MDL finds the optimal values of precision and conciseness of the trajectory partitioning based on Equation (5).

(5) $$MDL=\sum_{j=1}^{M-1}log2\left(len\left(p_{j}p_{j+1}\right)\right)+\sum_{j=1}^{M-1}\sum_{K=j}^{j+1}(log2\left(d_{⊥}\left(p_{j}p_{j+1},p_{k}p_{k+1}\right)\right)+log2\left(d_{\theta }\left(p_{j}p_{j+1},p_{k}p_{k+1}\right)\right)$$

where $d_{⊥}$ is the perpendicular distance, and $d_{\theta }$ is the angular distance between consecutive points. The objective of MDL is to find the characteristic points (i.e., the points where the movement pattern changes). To achieve this, the trajectory is segmented, and the MDL cost is calculated for each segment both before and after the proposed split. If the MDL cost is reduced, the corresponding point is accepted as a characteristic point, leading to further segmentation of adjacent parts of the trajectory. We used this algorithm to partition trajectories since it is well-suited for activity recognition with large datasets. This method is based purely on trajectory data and is fast. Moreover, it does not require labelling and does not highly depend on domain knowledge. However, this method’s accuracy is highly dependent on stop and movement pattern recognition and is sensitive to abrupt high-speed movements.

### 3.3. Real-Time Trajectories Corrector

The spatial and attribute accuracy of trajectories significantly impacts the accuracy of complex events. Therefore, GICEDCAM should address and fix trajectory data in real-time before it enters the event-matching step. There are two major issues in analyzing moving object trajectories. The first challenge pertains to incorrect tracking identification due to occlusions or disjoint cameras’ Field of View (FOV). This issue is addressed by the tracking re-identifier component in Section 3.3.1. The second issue involves noise and disturbances in trajectory data due to random errors in object detection or inconsistencies between the camera inputs and other sensors’ observations. Section 3.3.2 introduces the trajectory of the spatial enhancer as a component to fix this issue.

#### 3.3.1. Tracking Re-Identifier

To detect complex events in a network of smart cameras and sensors, we need to track objects across different camera FOVs (i.e., inter-camera tracking). Additionally, object occlusions, object detection false negatives, or frame light inconsistencies might cause gaps in moving object tracking (i.e., intra-camera tracking). DeepSORT is a widely used algorithm that operates in both inter- and intra-camera tracking, has low complexity, and delivers high accuracy for both small and large-scale datasets [59]. However, DeepSORT remains vulnerable to appearance variations and is sensitive to detection errors [60]. This means that if an object is not detected for several consecutive frames, DeepSORT concludes that the object is no longer present.

**Figure 5:**
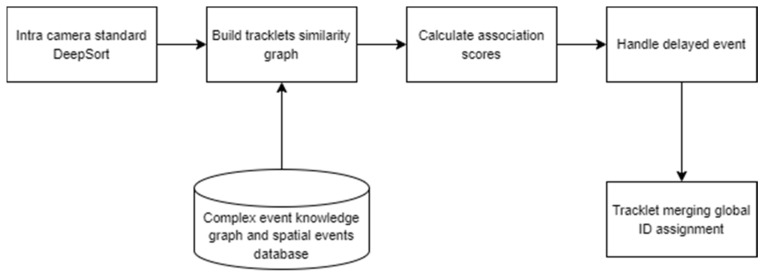
Event-aware DeepSORT workflow.

In some cases, considering the continuity of spatial events (e.g., a person holds a bottle) based on the KG pattern would help match tracklets more efficiently. For example, three people left the room, which is monitored by camera A, and one of them held the bottle, and all of them left the room at the same time, while camera B captured a person holding a bottle. Then the continuity of the “holding a bottle” spatial event can be evidence that the trajectories seen in camera A and camera B belong to the same person. Therefore, we propose a modified DeepSORT algorithm (event-aware DeepSORT) that integrates the semantics of spatial events into the tracking process. This enhancement aims to improve both the performance and accuracy of object tracking. Figure 5 illustrates the linear workflow of the event-aware DeepSORT algorithm.

In the first step, for each camera, moving objects are tracked, and local tracking IDs are assigned to them using the standard DeepSORT algorithm. Standard DeepSORT tracks objects using appearance embeddings, motion, and timestamped features. In the next step, a similarity graph of tracklets is built based on the appearance, motion, and spatial event features of tracklets. Spatial events are detected and assigned to tracklets using the spatial event detector component. In the similarity graph, nodes are tracklets from all cameras, and edges are built based on the affinity scores (i.e., association score), which are calculated based on Equation (6).

(6) $$S_{total}=\lambda_{1}S_{appearance}+\lambda_{2}S_{motion}+\lambda_{s}S_{event}\;,\;S_{total}\in [0,1]$$

S_apperance_ is the cosine similarity of the appearance features of tracklets. To calculate S_apperance_, the appearance of the tracklets is stored in two vectors *f_1_* and *f_2_*. We can consider the last *k* tracklets average for more robust tracklet matching. Equation (7) provides more details on how the appearance score is calculated.

(7) $$S_{appearance}(f_{1},f_{2})=\frac{f_{1}{.f}_{2}}{\left\|f_{1}\right\|\left\|f_{2}\right\|},\;f_{i}=\frac{1}{k}\sum_{j=1}^{n}f_{ij}$$

In Equation (6), S_motion_ is calculated based on the estimated time and the position of moving objects between tracklets. DeepSORT uses the Kalman Filter (KF) to predict the next tracklet location. The S_motion_ is calculated based on Equation (8). In this equation, *d_M_* is the Mahalanobis distance, *x* is the measurement (bounding box center) of the current detection, $\hat{x}$ is the predicted mean, and *S* is the predicted covariance matrix from the KF.

(8) $$S_{motion}=\exp\left(-\frac{1}{2}d_{M}\left(x,\hat{x}\right)\right),\;{\;\;d}_{M}\left(x,\hat{x}\right)=\sqrt{{\left(x-\hat{x}\right)}^{T}S^{-1}(x-\hat{x})}$$

In Equation (6), S_event_ is 1 if two tracklets share the same expected event (e.g., “*person holds bottle*” before and after the transition), 0 otherwise. $\lambda_{1}$, $\lambda_{2}$, and $\lambda_{3}$ are hyperparameters and are set based on the reliability or expectations of scores. For example, if spatial events are well annotated, $\lambda_{3}$ receives higher weight/values.

Sometimes, the spatial event is detected in the first tracklet of the second trajectory. For example, the spatial event (e.g., person picks the bottle) is not detected as the first observed tracklet in camera B. Therefore, we need to reevaluate past edges of the tracklet graph if a spatial event (e.g., a person picks up a bottle) is detected in the middle of the second trajectory. Prior to the detection of this spatial event, the scores of the candidate tracklet graph edges were relatively low. Therefore, it is necessary to update these candidates’ edge scores considering the newly detected spatial event. Equation (9) illustrates the procedure for updating the scores of candidate edges, where $S_{candidate}$ is the past candidate edge score, γ denotes the confidence boost scale for delayed events, α is the decay rate that indicates how much trust is given to earlier frames, and t and t’ represent the times of the detected spatial event and the candidate tracklet.

(9) $$S_{updated}=S_{candidate}+\gamma S_{delayed},\;{\;\;\;S}_{delayed}=e^{-\alpha (t^{\prime }-t)}$$

In the last step, we aim to optimize the graph edges, merge tracklets, and assign global IDs to tracklets. To do so, we prune the graph edges based on a predefined threshold, which represents the expected level of confidence in the connections between tracklets. Subsequently, we optimize the graph by merging tracklets and assigning global IDs through the use of a Hierarchical Clustering (HC) approach, a greedy matching algorithm that finds the best tracklet matches [61]. It employs the uses an agglomerative approach, merging the most similar tracklets and stopping when inter-cluster similarity falls below the threshold. This threshold can be determined through empirical tuning, starting with a default value that can be adjusted based on the specific characteristics of the dataset and application requirements.

#### 3.3.2. Trajectory Spatial Enhancer

GICEDCAM uses various data sources, such as camera streams, IMU, or GPS, to detect complex events. These data sources are ingested at different rates and levels of accuracy. This inconsistency of rates and accuracy is a potential source of trajectory noise. For example, the GPS data rate is 1 (Hz) while the camera stream rate is 30 fps (30 Hz). This means we expect denser trajectories from cameras, whereas GPS trajectories are sparser. Another issue is the objects’ bbox jittering due to the small changes in detection confidence or Non-Maximum Suppression (NMS) jittering. The jittering causes noise in the moving object’s trajectory.

**Figure 6:**
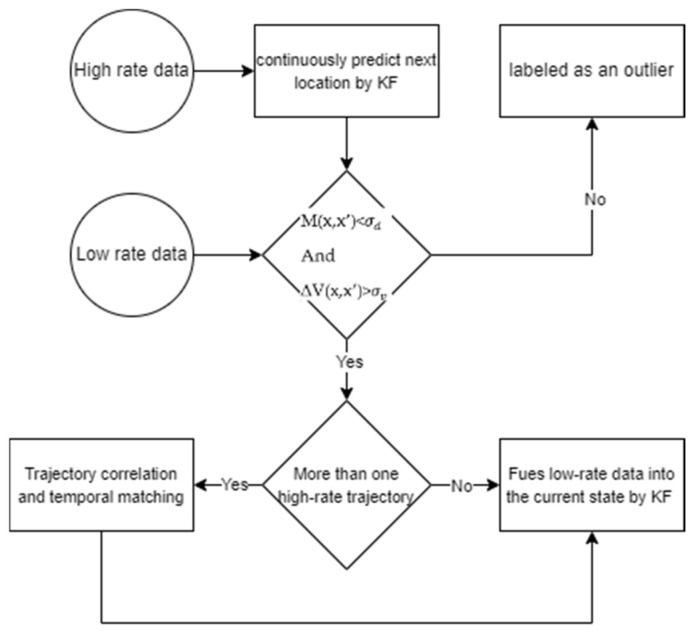
Workflow of enhancing trajectory data.

To solve the issue of integrating low-rate and high-rate trajectory data, we can apply upsampling of low-rate data or downsampling of high-rate data. Downsampling high-rate data would improve the performance of detecting spatial events, but it would reduce confidence in tracking and matching tracklets. On the other hand, upsampling low-rate data would increase the accuracy of tracking re-identification, but it reduces the performance of high-level event detection, and it is vulnerable to the low accuracy of low-rate data. Thus, we keep all data and update it in real-time. Figure 6 illustrates the workflow of the trajectory data correction procedure. In this workflow, high-rate data locations are continuously predicted using the KF algorithm, a widely used algorithm for the prediction and estimation of location data [62]. In the next step, for each high-rate trajectory, the predicted value (*x’*) is compared to the current low-rate value (*x*) based on the velocity difference *∆V* and Mahalanobis distance *M*. If they exceed the threshold values (i.e., $\sigma_{d}$ and $\sigma_{v}$), they are considered outliers and removed from the trajectory data. If more than one high-rate trajectory is found to match the low-rate data, temporal matching is triggered. It checks which trajectory the past k low-rate points matched. It then chooses that trajectory for fusion; otherwise, the algorithm proceeds to the fusion step with all candidates. In this step, the predicted value and current state values are used in the KF to update the low-rate data. As a result, high-rate trajectory data are refined, and low-rate data drift is corrected.

## 4. Implementations, Results, and Discussions

### 4.1. Data

**Figure 7:**
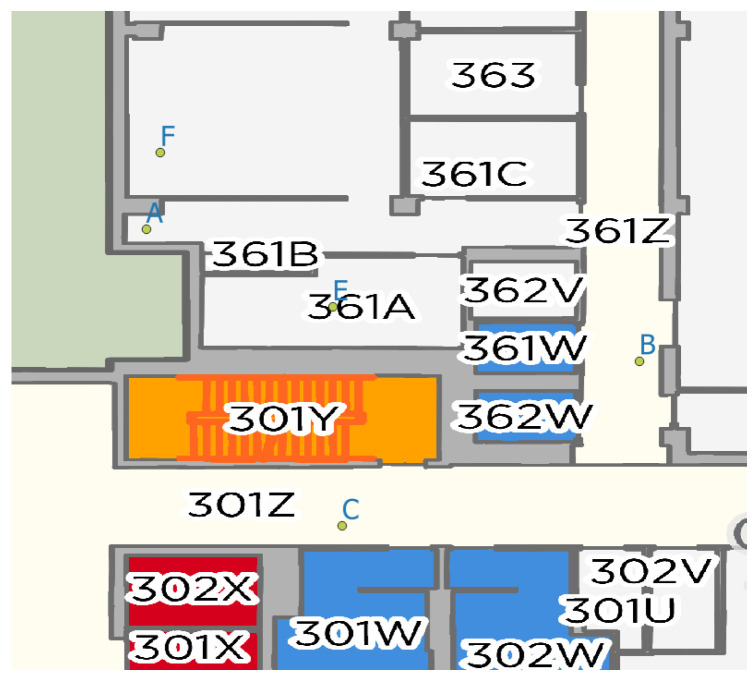
Camera locations in the CCIT building.

To evaluate the proposed framework, we used the data recorded by the GeoSensorWeb lab (The GeoSensorWeb lab is one of the University of Calgary labs that focuses on Geospatial data analysis and IoT) team. The data comprises 14 video streams captured by three smartphones from five distinct locations in the Calgary Center of Innovative Technologies (CCIT) building. The map of the building, including the camera points (A, B, C, E, and F), is illustrated in Figure 7. The Sony Xperia 10 III XQ-BT52 camera (made in Pathum Thani, Thailland) is in location A. The camera of the Apple iPhone 11 Pro Max (made in Zhengzhou, China) is in locations B and E. The camera of the Apple iPhone 14 Pro (made in Zhengzhou, China) is located on locations F and C.

We also used Bosch Sensortec 6-axis IMU sensor data for one person acting as a moving object, along with signal strength data from three access points corresponding to the person in the scenarios. In total, this amounts to four datasets, comprising both IMU and Wi-Fi signal strength measurements. IMU data includes accelerometer, gyro, and orientation readings of moving objects and cameras. We use the sensor readings as inputs to the GICEDCAM framework.

**Figure 8:**
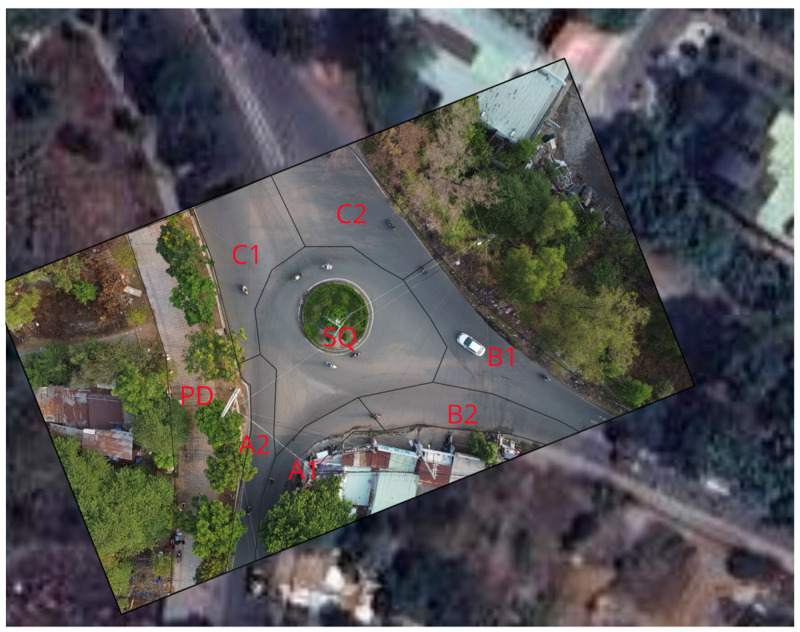
The zones map of the roundabout right-of-way violation scenario. A1, and A2 are the streets located in the south of the roundabout SQ. B1, and B2 are the sreets crossing the east of the roundabout SQ. C1, and C2 are located in the north of the roundabout SQ.

To evaluate the proposed framework under higher object loads and more complicated scenarios, we used the UIT-Adrone dataset [63]. The dataset includes 12 videos taken of a roundabout, which is located in the International University—VNU-HCM community area. The videos were recorded by a drone and captured cars, motorbikes, motorcycles, tricycles, bicycles, buses, trucks, vans, and pedestrian movements in the roundabout and the connected streets. We trained a YOLOv8 model on the vehicle data with eight classes. Details of the training results are available in the UIT-Drone-YOLO Kaggle repository, and the trained model is available in the corresponding model repository (https://www.kaggle.com/models/sepehrhonarparvar/uit-adrone-yolo accessed on 1 August 2025). We considered the roundabout right-of-way violation as a complex event. Figure 8 shows the roundabout map used in our evaluation.

Three scenarios are acted on and captured by smartphone cameras, and one scenario is defined for the roundabout right-of-way violation, which is summarized in Table 2. To illustrate how GICEDCAM detects complex events, we provide demo videos for Scenario 2 (Video S1) and Scenario 3 (Video S2) in the Supplementary Materials.

### 4.2. GICEDCAM Framework Implementation

**Figure 9:**
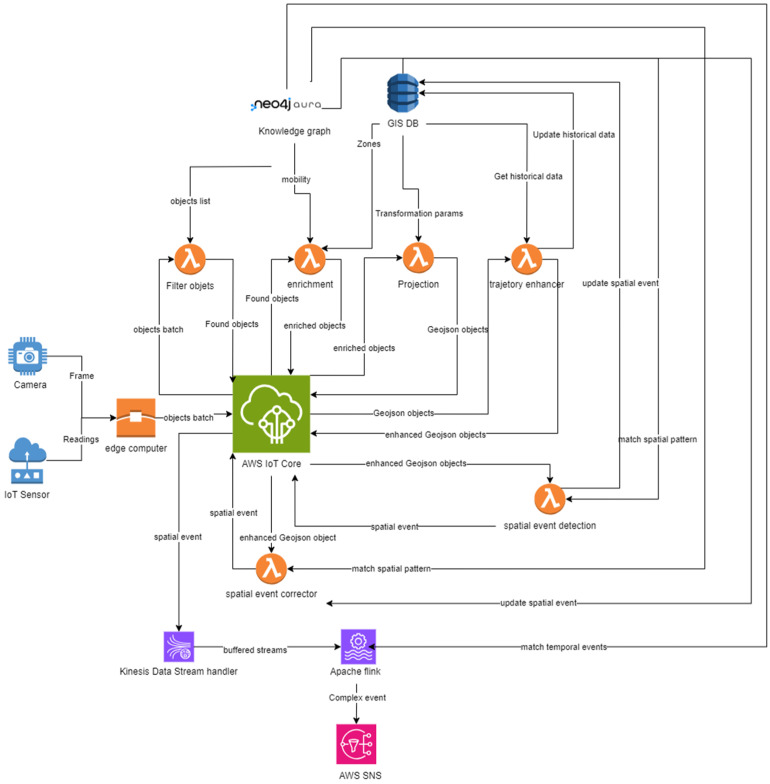
Implemented GICEDCAM data pipeline architecture.

To evaluate GICEDCAM’s performance against traditional CED frameworks, we designed and implemented the GICEDCAM data pipeline as shown in Figure 9. The camera streams and sensor readings are fed into a PC with an Intel Core i7 CPU and an NVIDIA GeForce RTX 2070 GPU. In this node, objects are detected, tracked, formatted, and published as Message Queuing Telemetry Transport (MQTT) messages to AWS IoT Core. Then, an Amazon Web Services (AWS) Lambda function is triggered to filter objects that are stored in a Neo4j Aura graph database. The results are published to AWS IoT Core to trigger the enrichment lambda. The enrichment lambda retrieves the geospatial zones from an AWS DynamoDB table and obtains the mobility status from Neo4j to enrich the objects. The enriched objects are published to the IoT Core to trigger the projection lambda function. This lambda takes the transformation parameters from DynamoDB and projects the objects onto a geospatial plane. The projected values are published to the IoT Core in GeoJSON format, serving as triggers for the appropriate processing functions for each object’s mobility status. If the mobility status indicates that the object is “moving”, the trajectory enhancer lambda function is activated. Conversely, if the mobility status indicates that the object is “fixed”, the spatial event detection lambda function is triggered. The trajectory enhancer lambda uses selected points from the historical trajectory to enhance the current moving object’s location. Then, the spatial event detection lambda is triggered once the trajectory enhancer lambda execution is completed. The spatial event detection queries spatial event patterns from the knowledge graph based on the detected objects and matches the found patterns with the processed spatial relationships. This function triggers the Spatial Event Corrector lambda. The Spatial Event Corrector lambda retrieves historical spatial events from DynamoDB and predicts whether any potential events are missed within the time window of events. This is the last lambda function that publishes corrected spatial events to the IoT Core. The IoT Core triggers the AWS Kinesis data stream handler to buffer spatial events and then passes them to Apache Flink to match temporal events based on reserved states, and publishes detected complex events to an AWS SNS notification service.

One of the most widely used video–CED frameworks is VIDCEP [15]. We use VIDCEP as the primary baseline because it is (i) open-source and end-to-end executable; (ii) allows online/streaming, supporting stateful spatial and temporal event operators that align with our task; and (iii) is domain-agnostic, enabling reproducible experiments with our detector outputs. Several recent CED systems discussed in our literature review either lack publicly released implementations or are specialized (e.g., domain- or operator-specific) and are thus not directly comparable within our streaming stack without substantial re-implementation, which would confound fairness and exceed the scope of this paper. We deployed VIDCEP to an AWS EC2 instance (four vCPUs, 16 GB RAM). We considered latency under load, end-to-end latency, and CPU and memory usage to compare the scalability, latency, and cost efficiency of GICEDCAM with VIDCEP. Edge node: Intel i7-10700, 32 GB RAM, RTX-2070. Cloud: AWS Lambda (Python 3.10, 512 MB, timeout 2 s, provisioned concurrency 5 for the corrector), AWS IoT Core (MQTT), DynamoDB (zones/calibration), Neo4j Aura (v2.0), Kinesis Data Streams (1 shard), Apache Flink (v1.0), parallelism 2. We use event time processing with allowed lateness *L* = 2.0 s and query window *W* = 11 s; complex events are finalized when *t*_end_ ≤ *ω* = max(event_time) − *L*. The data batches between the edge and stateless layer are JSON Lines (JSONL) format and the Objects schema. The data batches between stateless components are in GeoJSON Lines format and the Projected Objects schema. The data batches between the stateless and stateful layers are in the JSONL format and follow the Events Schema. The data schemas are illustrated in Appendix B. For the Spatial Event Corrector, we used the following configurations:

corrector :

c method: TRAJ    # BN | LSTM | TRAJ

 delta_probe : 0.3

 epsilon_time : 0.3

 budget_ms : 30

 BN :  [ threshold : 0.70 ]

 LSTM : [ weights_uri : s3://…/lstm.pt , window : 32 , stride : 1 , threshold : 0.80 ]

 TRAJ : [ dtw_radius : 5 , beta : 0.6 , threshold : 0.90 , roi_uri : s3://…/rois.json ]

Latency under load is defined as the wall-clock time from the first object written to the KG to the emission of the corresponding complex event while increasing concurrent stream fan-out N∈(1, 2, 4, 8, 16, 20). We simulate load by replaying a single video N times in parallel and publish detections after the edge node to isolate downstream ingestion and event processing; detectors/trackers, queries, window W = 11 s, and hardware are held constant. Table 3 reports the mean latency for each N.

Results demonstrated that the latency under load for GICEDCAM was similar up to eight simultaneous video streams. The latency difference between GICEDCAM and VIDCEP increased dramatically when processing eight or more streams. GICEDCAM broke down spatial event detection into smaller stateless processes. The use of parallel, event-driven serverless functions allows GICEDCAM to process high input rates without severe performance degradation, making it better suited for large-scale, multi-camera deployments. This architecture increased the horizontal scalability of the data pipeline. It means that GICEDCAM performs better under higher loads, and it is more suitable for large Internet of Smart Cameras (IoSC) networks. On the other hand, VIDCEP could be a good candidate for small networks.

Table 4 includes the latency measurements of GICEDCAM and VIDCEP for all four scenarios. To test end-to-end latency, we measured the sum of all processing times in the time window that starts from the time that the first object of a complex event is detected and ends with the time that the complex event is detected. This time is measured by reading AWS CloudWatch logs. For GICEDCAM, it is the total execution time of all processing nodes (i.e., lambda functions, Kinesis Data Stream handler, and Apache Flink), and, for VIDCEP, it would be EC2 execution time. We measured the end-to-end latency for scenarios one, two, three, and four.

Across all t. scenarios, GICEDCAM reduced end-to-end processing time by approximately 30% relative to VIDCEP, with the *p*-value (0.0029) confirming the statistical significance of this improvement. The effect size (Cohen’s d = 10.68) is exceptionally large, indicating that the latency reduction is not only statistically robust but also operationally impactful for time-critical applications. These gains can be attributed to the decoupled, event-triggered processing pipeline and optimized data flow in GICEDCAM. Also, as the number of objects per frame grows, the latency gap between GICEDCAM and VIDCEP widens. This difference is due to the parallel processing of objects’ row batches and breaking down complicated queries into simple calls in GICEDCAM nodes.

To measure computational cost, we measured CPU and memory usage of the entire data pipeline over the interval from the first to the last frame. CPU and memory usage are read by AWS CloudWatch logs. For GICEDCAM, it is the total CPU and memory usage of lambda functions, Kinesis Data Stream, and Apache Flink, and for VIDCEP, it is the EC2 CPU and memory usage for the time window. Table 5 illustrates the log measurements for GICEDCAM and VIDCEP. The results showed that GICEDCAM used less memory and CPU for the entire end-to-end CED. GICEDCAM’s memory usage is 59% lower than VIDCEP’s, and GICEDCAM’s CPU usage is 36% lower than VIDCEP’s. In total, GICEDCAM is about 48% cheaper than VIDCEP. Breaking down spatial event detection and correction into smaller stateless components helped us use memory and CPU more efficiently. In other words, a large portion of computations was handled by AWS Lambda functions, which utilize computational resources only when needed. The results also demonstrated that the VIDCEP computational cost is significantly higher for longer camera streams and a larger number of objects.

**Figure 10:**
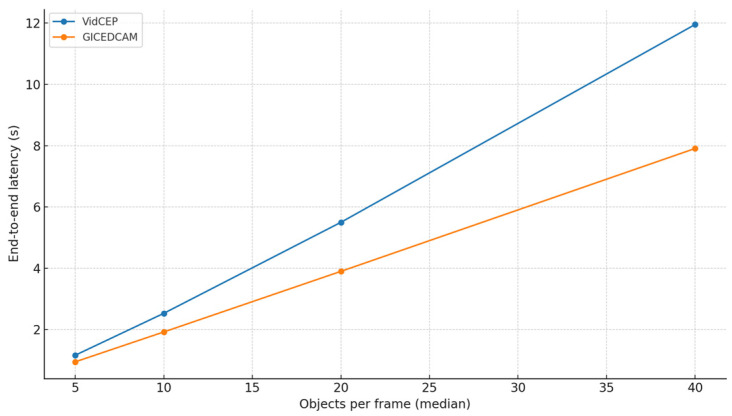
Latency values of GICEDCAM and VIDCEP vs. objects per frame.

**Figure 11:**
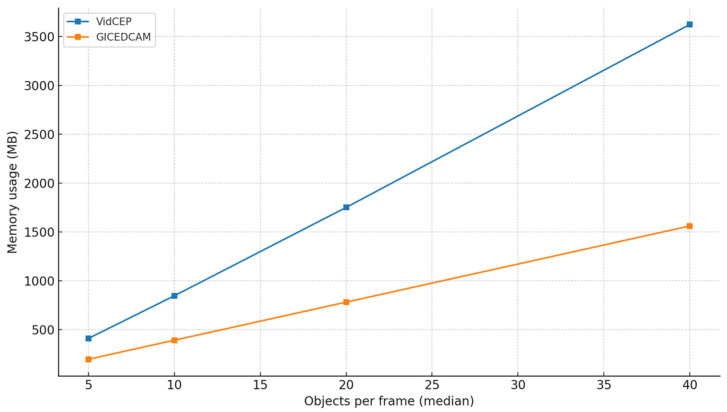
Memory usage values of GICEDCAM and VIDCEP vs. objects per frame.

To probe generality beyond a single baseline, we introduce objects per frame (OPF) scaling on Scenario 3. We vary the median detections per frame over {5, 10, 20, 40} using controlled fan-out/thresholding while holding videos, frame rate, temporal window (8 s), queries, and hardware constant. Figure 10 and Figure 11 illustrate OPF impacts on the latency and memory usage as OPF curves for GICEDCAM and VIDCEP.

Figure 10 and Figure 11 illustrate end-to-end latency and peak memory versus OPF for GICEDCAM and VIDCEP. Across all loads, GICEDCAM is faster and more memory-efficient, and the advantage grows with OPF. At 5→10→20→40 OPF, the latency reductions are 18.6% → 24.0% → 29.1% → 33.8%; memory reductions are 52.2% → 53.9% → 55.4% → 56.9%. Over this range, both systems scale approximately linearly with OPF (R^2^ ≥ 0.999), but VIDCEP’s slope is markedly steeper (latency 0.310 s/OPF vs. 0.199 s/OPF; memory 92.1 MB/OPF vs. 39.0 MB/OPF). This indicates that GICEDCAM’s stateless filtering and distributed processing keep growing closer to linear with a lower cost per additional object, leading to an increasing margin at higher scene densities.

### 4.3. Spatial Event Correction

**Figure 12:**
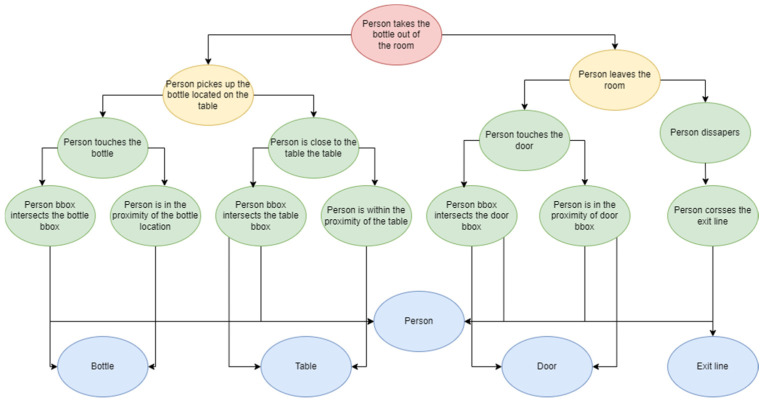
Knowledge graph of the Scenario 2 complex event.

One of the most important components of the GICEDCAM framework is the Spatial Event Corrector. To evaluate this component, we used 8 video streams of Scenario 4. The targeted missing event is the “person picks the bottle”, and the knowledge graph of the Scenario 2 complex event is shown in Figure 12. In the figure, the red oval is a complex event, the yellow ovals are the temporal events, the green ovals are the spatial events, and the blue ovals are the objects.

The objective is to evaluate the accuracy and performance of detecting the missing event for the BN method, LSTM, and trajectory analysis approach. For accuracy, we compared the false positives and false negatives of all three methods. Table 6 includes the counted precision, recall, and F-score for the BN approach, LSTM, and trajectory analysis method.

To evaluate the performance of the approaches, we used the processing time for the Spatial Event Corrector for all three methods over all four scenarios. Table 7 provides the average processing time of the three spatial event correction methods over all four scenarios.

A comparison between the results of spatial event correction methods demonstrated the highest value of recall for LSTM and the lowest for the BN approach. This means that LSTM is the most reliable method in cases where we do not want to miss any spatial event. It is better to use LSTM and trajectory analysis approaches when we do not want to let any incorrect spatial event come into the CED process. F-score values proved that LSTM delivers the highest precision and recall together in total. The BN approach provides the lowest latency and can be easily fitted to the GICEDCAM architecture. Results demonstrated that the complexity of the complex event KG impacts the latency values, but LSTM is the most affected method. In general, the trajectory analysis approach provides moderate and high accuracy and low latency in detecting missing spatial events, and it is well-fitted to the GICEDCAM architecture. However, the results also demonstrated the greater drop in recall for Scenario 3, which means the higher sensitivity of the proposed trajectory-based approach against a larger number of objects.

### 4.4. Trajectory Corrector

To evaluate the tracking re-identifier component, we tested the trajectory of Scenario 2 with a single moving object, Scenario 3 with multiple moving objects, and Scenario 4 with intentional occlusions. The trajectory points global IDs are labelled manually as ground truth. We considered precision as the main criterion to evaluate the tracking re-identifier module since the most important error of tracking algorithms is incorrect tracking ID assignment (i.e., false positives). Table 8 includes the latency and precision values of the proposed event-aware DeepSORT for the three scenarios. We also measured the statistical significance, such as *p*-value and effect size (Cohen’s d). The precision comparison between event-aware DeepSORT and standard DeepSORT yields a *p*-value of 0.098, which is above the 0.05 significance threshold, indicating that the observed improvement is not statistically significant. However, the Cohen’s d of 1.03 represents a large effect size, suggesting that the precision gains are practically meaningful, even if the small sample size prevents reaching statistical significance. For latency, the *p*-value of 0.0399 is below 0.05, showing a statistically significant increase in processing time when using event-aware DeepSORT. The Cohen’s d of 1.43 corresponds to a very large effect size, confirming that the latency increase is substantial in practice. This indicates a trade-off: event-aware DeepSORT offers large precision gains (practically speaking) but also significantly higher latency compared to standard DeepSORT.

**Figure 13:**
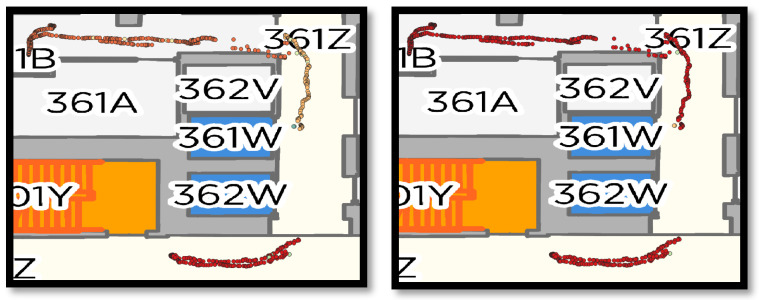
Person trajectories classified by global tracking_id: (**left**) before applying event-aware deep sort. (**right**) after applying event-aware deep sort.

We also visualized the person trajectories before and after applying the proposed event-aware DeepSORT in Figure 13. The points are classified and colored by the global tracking_IDs.

The results show that the Trajectory Corrector component has a direct effect on CED accuracy. Any discontinuity or incorrect tracklets would cause false negatives and false positives in CED. However, the effect is obvious, and it is not essential to evaluate or test the impact of trajectories on CED. Therefore, in this section, we evaluate tracking the re-identifier component in terms of precision and latency. The proposed event-aware DeepSORT improved tracking precision across all tested scenarios compared to standard DeepSORT, with practical significance supported by a large effect size (Cohen’s d = 1.03). Although the *p*-value for precision (0.098) did not reach statistical significance—likely due to limited sample size—the latency increase (*p* = 0.0399, d = 1.43) was both statistically and practically significant. This confirms a trade-off: while event-aware DeepSORT enhances tracking quality, it also introduces measurable processing overhead. Finally, Figure 10 shows that most of the false positives belong to the points which are farther from others. It means that, in Equation (6), the spatial event hyperparameter has a lower value than the motion hyperparameter. Consequently, motion velocity and distance between points are more impactful than spatial event reasoning.

## 5. Conclusions

GICEDCAM is a geospatial, multi-layer streaming pipeline for complex event detection that reorders the conventional workflow. Stateless KG-gating and projection standardize detections before any stateful operators, and a gap-triggered Spatial Event Corrector imputes missed predicates using one of BN/LSTM/trajectory methods. A finite-state (NFA) matcher then composes temporal and complex events under explicit event-time watermark and window policies. The design targets fixed cameras with static homographies and exchanges data in GeoJSONL; our implementation details the KG schema, operators, and configuration needed for reproducibility.

On our testbed, GICEDCAM delivers lower latency and resource use than VIDCEP, the only open-source, end-to-end baseline aligned with our ODSM setting. Scenario evaluations show an average ≈30% reduction in end-to-end latency. Under objects per frame (OPF) scaling from 5 to 40, both systems remain roughly linear, but GICEDCAM exhibits smaller slopes, indicating a lower per-object cost that widens the margin at higher scene densities. For spatial event correction, LSTM attains the highest recall/F1 at higher latency, BN is fastest with lower recall, and the trajectory method offers a practical accuracy–latency trade-off that fits the stateless design.

This study’s scope is limited to one indoor multi-camera environment and a UIT-Drone roundabout case, assumes fixed cameras with static calibrations, and compares primarily against VIDCEP due to the scarcity of reproducible recent frameworks. Future work will broaden evaluation to additional public benchmarks and diverse conditions (lighting, weather, crowding), incorporate new open-source baselines as they become available, and examine dimensions such as query expressiveness, optimizer behavior, and explainability, and extend ablations (e.g., window/ω sensitivity, corrector selection policies). We also plan to investigate calibration-drift handling and, longer-term, adaptations to moving/unstable cameras.

## Figures and Tables

**Table 1:** ODSM-based CED frameworks’ strengths and limitations.

Framework	Event Matching Strategy	Strengths	Limitations
**EventNet**	Video ontology linking object relationships to complex event concepts; semantic querying	Structured semantic representation; supports ontology-driven search	Requires manual ontology creation; limited scalability for real-time multi-camera networks
**Trajectory-based Models (e.g., hypergraph pairing)**	Assign event semantics through trajectory pairing; cluster-based abnormal behavior detection	Good for motion-based behavior recognition; captures trajectory semantics	Sensitive to tracking errors; limited handling of static object interactions
**Hierarchical Models**	Multi-layered feature aggregation from frame to temporal concepts	Reduces error propagation; modular event detection	overfits on relatively simple events, leading to lower performance than certain baseline methods
**VideoStorm**	Lag- and quality-aware query processing	Resource-efficient allocation; adaptable query performance	Focused on query performance, not false positive/negative reduction
**BLAZEIT**	FrameQL-based query optimization over DNN inference	Lowers DNN computation cost; selective frame analysis	Limited to simpler patterns; less effective for multi-camera spatial relationships
**Logical Reasoning Hybrid**	Combines logical reasoning with simple event detection	Enhances semantic interpretation; flexible rule creation	Higher computation cost; less optimized for large-scale streaming
**VIDCEP**	ODSM with VEQL and Video Event Knowledge Graph (VEKG)	Flexible spatiotemporal queries; domain-independent	Fully stateful → high computational cost; scalability issues with large object counts
**MERN**	Semantic-rich event representation; multi-entity relation network	Strong semantic modelling; supports multi-entity interactions	Relies heavily on the completeness and accuracy of domain ontologies, which may limit generalization to new domains; integration of DL models with semantic CEP adds system complexity
**NLP-guided Ontologies**	Uses NLP to enhance video event ontologies	Improved semantic matching; better generalization from text cues	Dependent on high-quality NLP models; limited spatial reasoning
**NOP**	Notification-Oriented Paradigm with chain-based queries	Efficient event chaining for specific domains	Restricted to certain event types; lacks general-purpose scalability

**Table 2:** Complex event detection test scenarios details.

#	Scenario	Camera Locations	Number of Videos
1	A person picks up the bottle from the chair in Room 361, then passes 361B corridor, then pours it into the mug located on the shelf in 361A	F, A, E	2
2	A person enters corridor 361B and picks up the bottle next to the printer. Then enter corridor 361Z and exit it. Then, enter 301Z and put the bottle on the bin door.	A, B, C	2
3	Roundabout right-of-way violation: A motorbike enters the roundabout SQ from street C1 before a car enters the roundabout SQ from street B1. Then, the car blocks the motorbike’s path and leaves the Square earlier than the motorbike.	International University—VNU-HCM roundabout	12
4	A person picks up the bottle from the table, moves to the door, opens the door, and exits corridor 361B. Every time a mandatory error is added to the spatial event (persons pick up the bottle). Occultation, losing track, and false negative object detection.	A	8

**Table 3:** Latency under load for GICEDCAM and VIDCEP.

Number of Video Streams	End-to-End Processing Time GICEDCAM	Latency Under Load GICEDCAM	End-to-End Processing Time VIDCEP	Latency Under Load VIDCEP
1	1.4 s	0 s	2.8 s	0 s
2	2 s	0.6 s	5.1 s	2.3 s
4	2.8 s	0.8 s	9.2 s	4.1 s
8	4.1 s	1.3 s	15.8 s	6.6 s
16	5.9 s	1.8 s	27 s	11.2 s
20	6.8 s	2.2 s	36.4 s	9.4 s

**Table 4:** End-to-end latency values for GICEDCAM and VIDCEP.

Scenario	GICEDCAM Latency	VIDCEP Latency
One	2.0 s	3.1 s
Two	2.9 s	4.2 s
Three	3.9 s	5.5 s

**Table 5:** GICEDCAM and VIDCEP CPU and memory usage values.

Scenario	GICEDCAM Memory (MB)	VIDCEP Memory (MB)	GICEDCAM CPU (%)	VIDCEP CPU (%)
One	540	1320	26	41
Two	670	1580	34	54
Three	780	1750	42	64

**Table 6:** Precision and recall of BN, LSTM, and trajectory-based approaches for spatial event correction.

Method	Precision	Recall	F-Score
BN	0.79	0.70	0.74
LSTM	0.86	0.87	0.86
Trajectory analysis	0.82	0.76	0.78

**Table 7:** Processing time values for BN, LSTM, and trajectory analysis spatial event correction methods.

Scenario	BN Method	LSTM	Trajectory Analysis
One	0.12 s	1.60 s	0.70 s
Two	0.14 s	1.65 s	1.1 s
Three	0.20 s	1.75 s	1.6 s
Four	0.10 s	1.42 s	0.51 s

**Table 8:** Latency and the precision values of the proposed event-aware DeepSORT for Scenarios 2, 3, and 4.

Metric	Scenario 2	Scenario 3	Scenario 4
Precision (DeepSORT)	91%	78%	70%
Precision (Event aware DeepSORT)	96%	85%	85%
Latency (DeepSORT)	0.15 s	0.25 s	0.25 s
Latency (Event aware DeepSORT)	0.22 s	0.37 s	0.40 s

**Figure A1:**
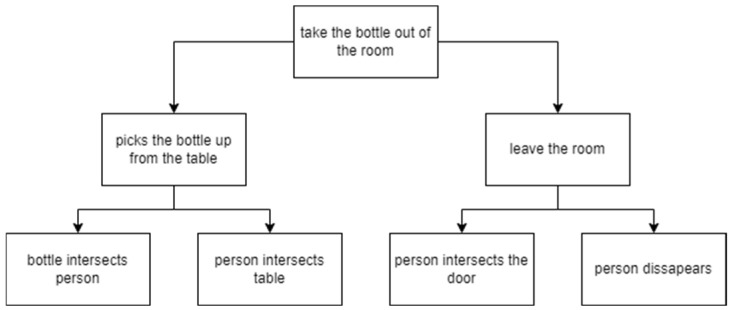
“Take the bottle out of the room” complex event knowledge graph.

**Figure A2:**
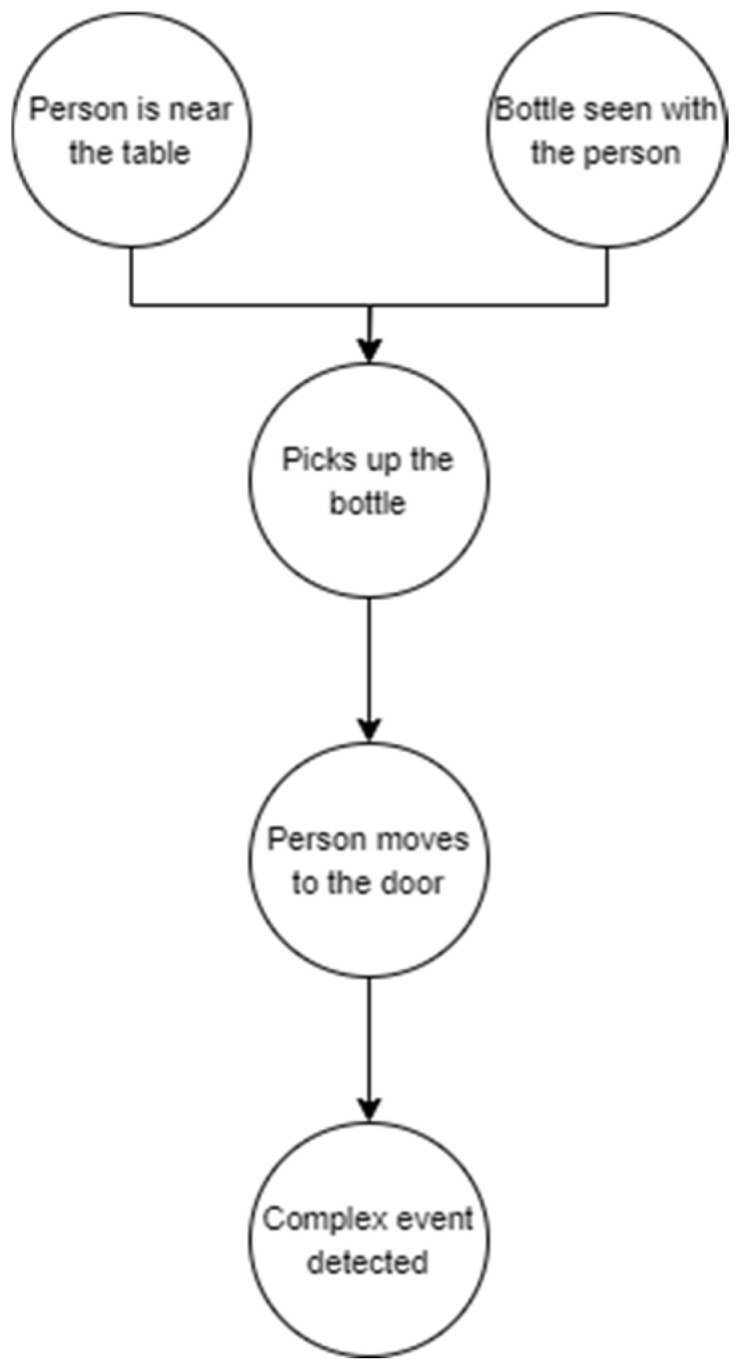
Bayesian Network for the person who takes the bottle out of the room complex event.

## Data Availability

The raw video data is available at https://zenodo.org/records/15817431, accessed on 26 August 2025.

## Supplementary Materials

The following supporting information can be downloaded at: https://www.mdpi.com/article/10.3390/s25175331/s1, Video S1: Demo video of complex event detection of scenario two, Video S2: Demo video of complex event detection of scenario three.

## Appendix A

### Appendix A.1

To demonstrate how BN identifies the missing spatial events, we examine the “take the bottle out of the room” complex event. In Figure 11, “person intersects bottle” and “person intersects table” as spatial events form the “picks the bottle up from the table” temporal event. The “person intersects the door” and “person disappears” spatial events form the “leaves the room” temporal event. The “picks the bottle up from the table” should happen before the “leaves the room” temporal event.

We assume that the person occluded the bottle and consequently went undetected at the moment the person picked it up. As a result, the spatial event “*bottle intersects person*” is missed, even though the system detected the event “person intersects bottle” shortly after “*person intersects table*”. This situation allows us to construct the BN graph as illustrated in Figure 12.

We want to calculate the probability of the “*picks up the bottle*” complex event given that the person nears the table and the bottle is seen with the person. We need to compute the probability of the missing event *P(Person Picks Bottle|Person Near Table = 1, Bottle Seen With Person Afterwards = 1)*. Based on the Bayesian theorem, Equation (A1) calculates the missing event probability.

(A1) $$P\left(PPB=1|PNT=1,BSP=1\right)=\frac{P(PPB=1,PNT=1,BSP=1)}{P\left(PNT=1,BSP=1\right)}$$

where PPB means Person Picks Bottle, PNT means Person Near Table, and BSP means Bottle Seen with Person afterward. Assuming Table 8 includes the probability values that experts have assigned as CPD values.

**Table A1:** Example probability values of CPD.

CPD Name	Probability
Bottle Seen With Person = 1	0.6
Person Near Table = 1	0.7
Complex Event Detected∣Person Moves To Door = 1	0.95
Person Moves To Door∣Person Picks Bottle	0.9
Person Near Table = 1, Bottle Seen With Person = 1	0.8
Person Near Table = 1, Bottle Seen With Person = 0	0.4
Person Near Table = 0, Bottle Seen With Person = 1	0.3
Person Near Table = 0, Bottle Seen With Person = 0	0.1

The probability of the missing spatial event (i.e., picking up the bottle) under conditions that the person was near the table and seen with the bottle afterward is equal to 0.8 × 0.7 × 0.6/(0.8 × 0.7 × 0.6+ 0.2 × 0.7 × 0.6) ~= 0.8.

### Appendix A.2

In the following example, we detect “*Picks up the Bottle*” as the missing spatial event.

In this case, we define LSTM variables as the tuple of person coordinates and spatial events at time t. Therefore, the input of the LSTM is defined in Equation (A2), where X_t_ and Y_t_ are the person’s coordinates and S_t_ is the spatial event.

(A2) $$X=[\left(X_{t-4},Y_{t-4},S_{t-4}\right),\left(X_{t-3},Y_{t-3},S_{t-3}\right),\left(X_{t-2},Y_{t-2},S_{t-2}\right),\left(X_{t-1},Y_{t-1},S_{t-1}\right)]$$

Equation (A1) defines the output label for the missing event. Here, LSTM learns from past movement patterns and infers whether the missing event happened.

(A3) $$Y_{t}=\mathrm{Person}\;\mathrm{Picks}\;\mathrm{Bottle}=[0,1]$$

We consider a five-time step per sequence to predict the missing event. Table A2 represents a numerical value for the five steps.

**Table A2:** Example of LSTM sequence input values.

Time	X	Y	Person Intersects Table	Bottle Seen with Person
t-4	3	5	0	0
t-3	3.5	5.5	0	0
t-2	4	6	1	0
t-1	4.5	6.5	1	0

Here, the bottle intersects with the person who is missing, but LSTM learns from past movement sequences and predicts the missing event. LSTM takes past movement sequences and predicts the missing event using Equations (A4) and (A5).

(A4) $$h_{t}=\sigma (W_{h}X_{t}+U_{h}h_{t-1}+b_{h})$$

(A5) $$Y_{t}=\sigma (W_{0}h_{t}+b_{0})$$

where *h_t_* is the LSTM hidden state at time *t*, *W_h_* (maps the input *X_t_* to the h_t_ state) and U_h_ (maps previously hidden state *h_t_*_−1_ to *h_t_*) are LSTM weight matrices, *Y_t_* is the probability of the missing event, *σ* is the Sigmoid activation function, and *b_h_* is the bias term. We use binary cross-entropy to predict the missing event.

(A6) $$L=-\sum_{i=1}^{N}\left[Y_{i}\log\left(\hat{Y_{i}}\right)+\left(1-Y_{i}\right)log(1-\hat{Y_{i})}\right]$$

The weights are initiated randomly and are updated during the training based on the Back Propagation Through Time (BPTT) algorithm, which is summarized in Equations (A7)–(A9).

(A7) $$W_{h}=W_{h}-\eta \frac{\partial L}{\partial W_{h}}$$

(A8) $$U_{h}=U_{h}-\eta \frac{\partial L}{\partial U_{h}}$$

(A9) $$b_{h}=b_{h}-\eta \frac{\partial L}{\partial b_{h}}$$

where η is the learning rate and ∂ represents the gradient of loss function over the weights and the bias term.

Assuming that the weight values output from the training procedure are as follows:

$$W_{h}=\left[0.2,\;0.3,\;0.4\right]\;U_{h}=\left[0.5,\;1\right]\;b_{h}=0.1$$

Then, the state value would be:

$$h_{t}=\sigma ((0.2\times 4.5)+(0.3\times 6.5)+(0.4\times 1)+(0.5\times 0.8)+0.1)$$

The output will be 0.54 if *W*_0_ is 0.7 and *b*_0_ is −0.5. This indicates the LSTM predicts a 54% probability that the person picks up the bottle at time t.

## Appendix B

### Appendix B.1. Object Schema

{

 “$schema” : “https://json-schema.org/draft/2020-12/schema” ,

 “$id” : “https://.../schemas/object.schema.json” ,

 “title” : “Object (raw detection)” ,

 “type” : “object” ,

 “additionalProperties” : false ,

 “required” : [ “object_type” , “camera_id” , “local_id” , “x_min” , ”y_min” , ”x_max” , ”y_max” , “detection_time” , “confidence” ],

 “properties” : {

  “object_type” : { “type” : “string” },

  “camera_id” :  { “type” : “string” , “description” : “Sensor/camera identifier” },

  “local_id” :  { “type” : “string” , “description” : “Per-camera track/detection ID” },

  “global_id” :  { “type” : “string” , “description” : “Cross-camera/global ID if known” },

  “x_min” :   { “type” : “number” , “description” : “X min of object bbox” },

  “y_min” :   { “type” : “number” , “description” : “Y min of object bbox” },

  “x_max” :   { “type” : “number” , “description” : “X max of object bbox” },

  “y_max” :   { “type” : “number” , “description” : “Y max of object bbox” },

  “detection_time” : { “type” : “number” , “description” : “Event-time (epoch seconds, float)” },

  “confidence” :   { “type” : “number” , “minimum” : 0 , “maximum” : 1 },

  “frame_id” :    { “type” : “integer” , “minimum” : 0 },

  “speed” :      { “type” : “number” , “description” : “estimated object speed by Yolo model” },

  “color_histogram” : {

   “type” : “array” ,

   “description” : “estimated object speed by Yolo model” ,

   “items” : {

    “type” : “number”

    }

   },

  “source” :     { “type” : “string” , “description” : “Producer tag (e.g., ‘edge-detector’)” }

 },

 “examples” : [

  {

   “object_type” : “person” ,

   “camera_id” : “A” ,

   “local_id” : “trk-0123” ,

   “x_min” : 642.5 ,

   “y_min” : 311.2 ,

   “x_max” : 670.3 ,

   “y_max” : 422.4 ,

   “ detection_time” : 1723645223.412 ,

   “confidence” : 0.92 ,

   “frame_id” : 1084 ,

   “color_histogram” : [ 2 , 0 , ... , 12 ],

   “speed” : 21.2

  }

 ]

}

### Appendix B.2. Projected Object Schema

{

 “$schema” : “https://json-schema.org/draft/2020-12/schema” ,

 “$id” : “https://gicedcam.org/schemas/projected-object.schema.json” ,

 “title” : “ProjectedObject (GeoJSON Feature)” ,

 “type” : “object” ,

 “additionalProperties” : false ,

 “required” : [ “type” , “geometry” , “properties” ],

 “properties” : {

  “type” : { “const” : “Feature” },

  “geometry” : {

   “type” : “object” ,

   “additionalProperties” : false ,

   “required” : [ “type” , “coordinates” ],

   “properties” : {

    “type” : { “const” : “Point” },

    “coordinates” : {

     “type” : “array” ,

     “minItems” : 2 ,

     “maxItems” : 2 ,

     “items” : { “type” : “number” },

     “description” : “[x, y] in meters, CRS = ‘cartesian-m’”

    }

   }

  },

  “properties” : {

   “type” : “object” ,

   “additionalProperties” : false ,

   “required” : [ “record_type” , “camera_id” , “local_id” , “class” , “detection_time” , “conf” , “mobility” , “crs” ],

   “properties” : {

    “camera_id” :  { “type” : “string” },

    “local_id” :  { “type” : “string” },

    “global_id” :  { “type” : “string” },

    “class” :    { “type” : “string” },

    “detection_time” : { “type” : “number” , “description” : “Event-time (epoch seconds, float)” },

    “mobility” :  { “type” : “string” , “enum” : [ “moving” , “fixed” ] },

    “zone_id” :   { “type” : “string” , “description” : “Assigned geospatial zone (if any)” },

    “mu” :     { “type” : “number” , “minimum” : 0 , “maximum” : 1 , “description” : “Fuzzy membership to zone_id” },

    “speed_m_s” :  { “type” : “number” },

    “traj_id” :   { “type” : “string” },

    “crs” :     { “type” : “string” , “const” : “cartesian-m” },

    “x_min” :   { “type” : “number” , “description” : “X min of object bbox” },

    “y_min” :   { “type” : “number” , “description” : “Y min of object bbox” },

    “x_max” :   { “type” : “number” , “description” : “X max of object bbox” },

    “y_max” :   { “type” : “number” , “description” : “Y max of object bbox” },

    “confidence” :   { “type” : “number” , “minimum” : 0 , “maximum” : 1 },

    “frame_id” :    { “type” : “integer” , “minimum” : 0 },

    “speed” :      { “type” : “number” , “description” : “estimated object speed by Yolo model” },

    “color_histogram” : {

     “type” : “array” ,

     “description” : “estimated object speed by Yolo model” ,

     “items” : {

      “type” : “number”

      }

     },

    “source” :   { “type” : “string” , “description” : “Producer (e.g., ‘projector’, ‘traj-enhancer’)” }

   }

  }

 },

 “examples” : [

  {

   “type” : “Feature” ,

   “geometry” : { “type” : “Point” , “coordinates” : [ 12.43 , 8.77 ] },

   “properties” : {

    “camera_id” : “A” ,

    “local_id” : “trk-0123” ,

    “global_id” : “P-00042” ,

    “class” : “person” ,

    “detection_time” : 1723645223.412 ,

    “confidence” : 0.92 ,

    “mobility” : “moving” ,

    “zone_id” : “361A-table” ,

    “mu” : 0.83 ,

    “speed_m_s” : 0.9 ,

    “traj_id” : “traj-007” ,

    “crs” : “cartesian-m” ,

    “source” : “projector” ,

    “x_min” : 642.5 ,

    “y_min” : 311.2 ,

    “x_max” : 670.3 ,

    “y_max” : 422.4 ,

    “detection_time” : 1723645223.412 ,

    “color_histogram” : [ 2 , 0 , … , 12 ],

    “speed” : 21.2

   }

  }

 ]

}

### Appendix B.3. Events Schema

{

 “$schema” : “https://json-schema.org/draft/2020-12/schema” ,

 “$id” : “https://../schemas/spatial-event.schema.json” ,

 “title” : “SpatialEvent” ,

 “type” : “object” ,

 “additionalProperties” : false ,

 “required” : [ “type” , “predicate” , “bindings” , “t” , “conf” , “corrected” ],

 “properties” : {

  “type” :   { “const” : “SpatialEvent” },

  “predicate” : { “type” : “string” , “pattern” : “^[A-Za-z][A-Za-z0-9_]*$” , “description” : “e.g., Near, InZone, Touches” },

  “bindings” : {

   “type” : “object” ,

   “description” : “Map from pattern variables to KG IDs (Object/Zone IDs)” ,

   “minProperties” : 1 ,

   “additionalProperties” : { “type” : “string” }

  },

  “t” :     { “type” : “number” , “description” : “Event-time (epoch seconds, float)” },

  “conf” :   { “type” : “number” , “minimum” : 0 , “maximum” : 1 },

  “corrected” : { “type” : “boolean” , “description” : “true if emitted by the Spatial Event Corrector” },

  “id” :    { “type” : “string” , “description” : “Deterministic record ID (optional)” },

  “source” :  { “type” : “string” , “description” : “Producer tag (e.g., ‘detector’,‘corrector’)” },

  “meta” :   { “type” : “object” , “description” : “Optional method metadata” , “additionalProperties” : true }

 },

 “examples” : [

  {

   “type” : “SpatialEvent” ,

   “predicate” : “Near” ,

   “bindings” : { “A” : “P-00042” , “B” : “Table-1” , “Z” : “361A” },

   “t” : 1723645224.105 ,

   “conf” : 0.91 ,

   “corrected” : false ,

   “source” : “spatial-detector” ,

   “id” : “se:Near:P-00042:Table-1:361A:1723645224.105”

  },

  {

   “type” : “SpatialEvent” ,

   “predicate” : “PickUp” ,

   “bindings” : { “A” : “P-00042” , “obj” : “Bottle-7” , “Z” : “361A” },

   “t” : 1723645225.330 ,

   “conf” : 0.86 ,

   “corrected” : true ,

   “source” : “corrector:TRAJ”

  }

 ]

}
